# Clinical and genetical heterogeneity of late-onset multiple acyl-coenzyme A dehydrogenase deficiency

**DOI:** 10.1186/s13023-014-0117-5

**Published:** 2014-07-22

**Authors:** Sarah C Grünert

**Affiliations:** 1 Center of Pediatrics and Adolescent Medicine, University Hospital Freiburg, Freiburg, Germany

**Keywords:** Glutaric aciduria type 2, Lipid storage myopathy, Muscle weakness, Metabolic decompensation, Riboflavin, ETFDH, ETFA, ETFB

## Abstract

**Background:**

Multiple acyl-CoA dehydrogenase deficiency (MADD) is an autosomal recessive disorder caused by deficiency of electron transfer flavoprotein or electron transfer flavoprotein dehydrogenase. The clinical picture of late-onset forms is highly variable with symptoms ranging from acute metabolic decompensations to chronic, mainly muscular problems or even asymptomatic cases.

**Methods:**

All 350 cases of late-onset MADD reported in the literature to date have been analyzed and evaluated with respect to age at presentation, diagnostic delay, biochemical features and diagnostic parameters as well as response to treatment.

**Results:**

Mean age at onset was 19.2 years. The mean delay between onset of symptoms and diagnosis was 3.9 years. Chronic muscular symptoms were more than twice as common as acute metabolic decompensations (85% versus 33% of patients, respectively). 20% had both acute and chronic symptoms. 5% of patients had died at a mean age of 5.8 years, while 3% of patients have remained asymptomatic until a maximum age of 14 years. Diagnosis may be difficult as a relevant number of patients do not display typical biochemical patterns of urine organic acids and blood acylcarnitines during times of wellbeing. The vast majority of patients carry mutations in the *ETFDH* gene (93%), while mutations in the *ETFA* (5%) and *ETFB* (2%) genes are the exceptions. Almost all patients with late-onset MADD (98%) are clearly responsive to riboflavin.

**Conclusions:**

Late-onset MADD is probably an underdiagnosed disease and should be considered in all patients with acute or chronic muscular symptoms or acute metabolic decompensation with hypoglycemia, acidosis, encephalopathy and hepatopathy. This may not only prevent patients from invasive diagnostic procedures such as muscle biopsies, but also help to avoid fatal metabolic decompensations.

## Background

Multiple acyl-CoA dehydrogenase deficiency (MADD, MIM #231680), also called glutaric aciduria type 2 (GA2), is an inborn error of metabolism affecting the oxidation of fatty acids as well as the catabolism of branched-chain amino acids, lysine and tryptophan. MADD is caused by deficiency of either an electron-transfer flavoprotein (ETF, encoded by *ETFA* and *ETFB*) or an electron-transfer flavoprotein dehydrogenase (ETFDH, encoded by *ETFDH*). Inheritance follows an autosomal recessive trait [[1]]. The metabolic defects result in impaired adenosine triphosphate (ATP) biosynthesis, excessive lipid accumulation in different organs and insufficient gluconeogenesis [[1]]. The clinical phenotype is heterogeneous and has been classified into three groups: neonatal onset with congenital anomalies (type 1), neonatal onset without anomalies (type 2), and mild and/or later onset (type 3) [[1],[2]]. While neonates usually present with severe metabolic decompensations including metabolic acidosis, non-ketotic hypoglycemia, hyperammonemia, hypotonia, coma and cardiomyopathy, the course and age at presentation of later-onset forms is extremely variable. In adolescents and adults, muscular or cardiac symptoms or episodic vomiting are usually first features suggestive for MADD [[1],[3]-[9]]. Therapeutic management mostly comprises a diet restricted in fat and protein and the avoidance of fasting. The majority of patients respond well to riboflavin therapy.

Diagnosis is based on both the urinary organic acid profile and the acylcarnitine pattern in dried blood/plasma. The characteristic urinary organic acid pattern comprises elevated levels of glutaric, ethylmalonic, 3-hydroxyisovaleric, 2-hydroxyglutaric, 5-hydroxyhexanoic, adipic, suberic, sebacic, and dodecanedioic acid without relevant ketonuria, especially if combined with glycine conjugates of C4 and C5 acids [[1]]. Acylcarnitine analysis usually reveals increased concentrations of several short-, medium- and long-chain acylcarnitines, such as C4, C5, C5-DC, C6, C8, C10, C12, C14:1, C16, C18:1 [[10]]. However, the diagnosis may be challenging in late-onset cases since the biochemical abnormalities may be mild, atypical or only detectable during metabolic decompensations. This report aims at providing a comprehensive description of late-onset MADD in its clinical and genetic heterogeneity by analysis of all cases published to date.

## Methods

A systematic literature search in PubMed was performed using the terms “multiple acyl-CoA dehydrogenase deficiency”, “glutaric aciduria type 2”, “glutaric acidemia type 2”, “ETFDH”, “ETFA” and “ETFB” in order to obtain comprehensive information on the clinical course of MADD patients with late onset of the disease (type 3). Late onset was defined as onset beyond the neonatal period (>28 days). The search was performed in January 2014. 350 cases of late-onset MADD published between 1979 and 2014 were identified (for detailed data and references see Additional file 1: Table S1). All cases were evaluated and analyzed with a special focus on the patients’ age at onset, age at diagnosis, symptoms, biochemical abnormalities, affected genes and response to riboflavin treatment. Riboflavin responsiveness was defined as a positive clinical response to treatment with improvement of symptoms, not only biochemical parameters.

## Results

**Figure 1:**
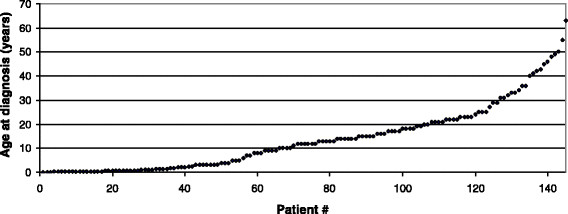
**Age at onset in 146 late-onset MADD patients.** Age at onset of symptoms ranges from early infancy to late adulthood. The mean age at onset of 146 patients with late-onset MADD of whom detailed information was given in the literature is 14.3 years. Of additional 56 cases reported by Wang et al. [[55]] and 71 cases reported by Xi et al. [[28]] only the mean age at onset (24.5 ± 12.6 years with a range from 4 to 55 years and 25.0 ± 13.3 years with a range from 4 to 36 years, respectively) is known. If these cases are also taken into account, the mean age at onset in this study cohort is 19.2 years.

350 cases of late-onset MADD were reviewed. Detailed information on all patients is displayed in the Additional file 1: Table S1 as well as in Table 1. 144 patients were female, 177 male, the sex of 29 individuals was not reported. Mean age at onset was 19.2 years (n = 273, range 0.13-68 years) (Figure 1). Seven patients were identified by newborn screening, and another 5 patients were diagnosed by early investigations in the neonatal period due to an affected sibling. In 2 patients the diagnosis was only made post mortem. Mean age at diagnosis of symptomatic patients was 17.6 years (n = 111, median 14.0 years, range 0.13-69 years). The mean delay between onset of symptoms and diagnosis was 3.9 years (n = 93, range 0-29 years). If only patients without acute decompensations were considered (n = 38), the mean latency was 3.7 years (range 0-29 years). 33.1% of patients (111/335) displayed acute signs and symptoms, i.e., metabolic decompensations with hypoglycemia and acidosis, while chronic features were reported in 85.3% (291/341) of patients. Chronic manifestations mainly comprised muscular weakness, exercise intolerance and muscle pain. 20.4% (68/333) had both, acute episodes and chronic symptoms. 5.2% (18/349) of patients had already died when reported in the literature (mean age at death 5.8 years, range 0.2-20 years). Among those only the minority (4/14, 28.6%) deceased during the initial metabolic crisis. In 5 of 11 patients with fatal outcome of whom detailed data were available, the diagnosis was known before the life-ending event. Three of those had been identified by newborn screening or early neonatal screening due to an affected older sibling while two patients had been diagnosed symptomatically in infancy/childhood. In the remaining 6 patients the diagnosis was only made during the fatal metabolic decompensation or post mortem. 2.6% (9/349) of patients have remained asymptomatic until a mean age of 6.15 years (0.5-14.0 years).

**Table 1:** Clinical information on 350 patients with late-onset MADD published in the literature

Sex	Female n = 144, male n = 177, not reported n = 29
Identified by newborn screening	2.0% (7/350) patients
Identified by family screening	1.4% (5/350) patients
Acute symptoms	33.1% (111/335) of patients
Chronic symptoms	85.3% (291/341) of patients
Acute and chronic symptoms	20.4% (68/333) of patients
Mean age at onset of symptoms	19.2 years (n = 273)
Mean diagnostic delay	3.9 years (0-29 years)
Asymptomatic	2.6% (9/349) of patients
Deceased patients	5.2% (18/349) of patients
Riboflavin responsiveness	98.4% (256/260) of patients

Results of organic acid analysis in urine were available in 258 patients. A typical metabolite pattern was detected at least once in 236/258 patients (91.5%) while only unremarkable organic acid results were reported in 22/258 individuals (8.5%). In some cases, elevated signals of only single metabolites such as ethylmalonic acid or glutaric acid were detected [[11]-[13]]. Some patients displayed a pathognomonic metabolite pattern only intermittently, i.e. during metabolic decompensations. In several patients, the initially detected biochemical abnormalities disappeared under treatment with riboflavin. Acylcarnitine profiles were at least intermittently characteristic for MADD in 201/214 patients (93.9%). In 13/214 individuals (6.1%) only normal acylcarnitine profiles were reported. Again, in some patients an abnormal acylcarnitine pattern was only found during metabolic decompensations or after a prolonged exercise test.

Among 260 patients of whom data on riboflavin therapy were available, 256 (98.4%) were reported to be clearly riboflavin-responsive, while in 2 patients efficacy of riboflavin therapy was only partial with merely “some improvement” of the muscular symptoms [[14],[15]]. In one of these patients riboflavin supplementation was discontinued later because of limited efficacy [[14]]. In one other patient in whom riboflavin therapy was started during severe metabolic decompensation fatal outcome could not be prevented [[16]]. Only one patient was reported to be clearly unresponsive to riboflavin [[17]].

**Figure 2:**
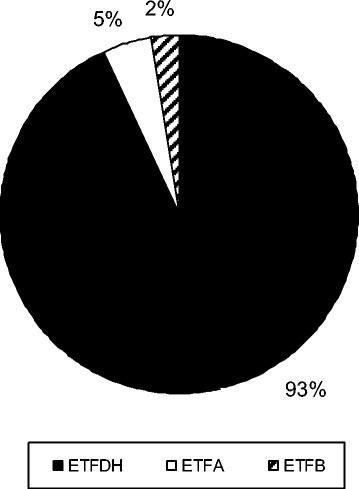
**Mutated genes in 245 patients with late-onset MADD.** The majority of late-onset MADD patients harbor mutations in the *ETFDH* gene. The frequency of *ETFDH, ETFA* and *ETFB* mutations in 245 patients with late-onset MADD is displayed.

The genotype was reported of 245 patients. The vast majority carried mutations in the *ETFDH* gene (228/245, 93.1%), while in only few patients *ETFA* and *ETFB* mutations were detected (11/245, 4.5% and 6/245, 2.4% of patients, respectively) (Figure 2). In one patient, no mutation was identified in the *ETFA* and *ETFB* genes, while the *ETFDH* gene was not studied. In 2 patients, no mutations could be delineated despite analyzing of the entire coding regions and flanking intronic sequences of the *ETFA, ETFB* and *ETFDH* genes [[18],[19]].

## Discussion

**Figure 3:**
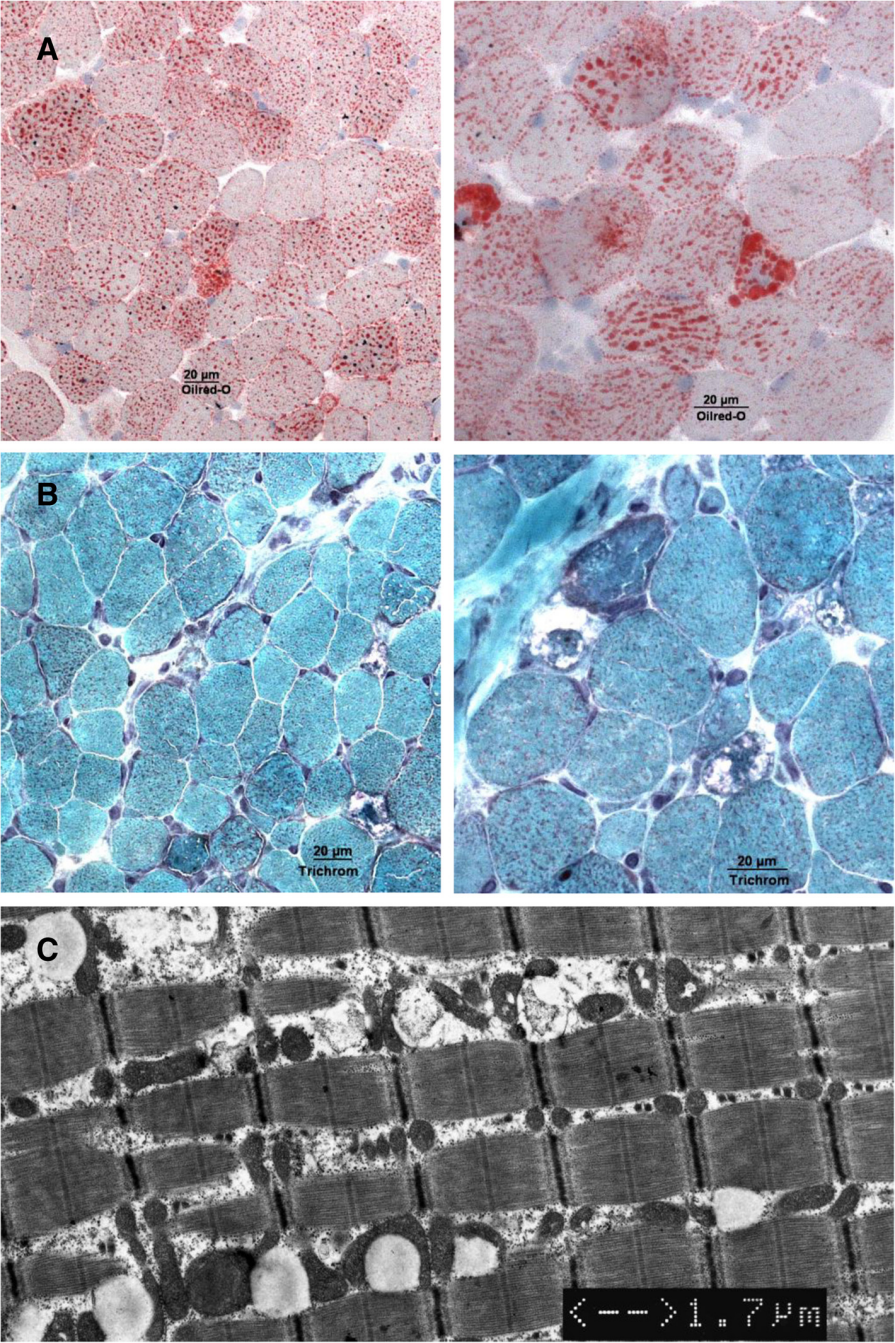
**Lipid storage myopathy.** Histological picture of lipid storage myopathy typical for late-onset MADD. **A** Oilred stain. **B** Trichrome stain. **C** Electron microscopy.

This evaluation of all published cases of late-onset MADD characterizes the clinical and genetic heterogeneity of this disorder with a special focus on typical symptoms, the diagnostic work-up and therapeutic options. The clinical phenotype of late-onset MADD is highly variable and ranges from acute, in some cases even fatal metabolic crises in infancy to asymptomatic adults. Even within the same family the clinical picture can vary profoundly [[20],[21]]. Although most patients become symptomatic within the first two decades, onset of symptoms ranges from the second month of life to late adulthood. The vast majority of patients presented with chronic, mainly myopathic symptoms. Nevertheless, one third of patients also displayed acute symptoms or metabolic decompensations which may be life-threatening if not treated adequately. Although these decompensations mostly occur in childhood, severe metabolic crises in adulthood have also been described [[14],[22]-[26]]. Decompensations are characterized by acidosis, hypoglycemia, elevated activities of transaminases, rhabdomyolysis with raised creatine kinase activity, and, eventually, hyperammonemia. These episodes are usually triggered by catabolic states, either due to infections and febrile illnesses or to a reduced energy supply. However, in adulthood, other triggering factors like operations, low-energy diets or weight loss of other reasons, alcohol, valproate therapy, pregnancy or labor may also play a role [[9],[15],[24],[27]]. Chronic features include exercise intolerance, muscle weakness, muscle wasting and muscular pain. Some cases may develop respiratory insufficiency [[13],[23],[28]], while rhabdomyolysis is not often seen. Muscle biopsies usually reveal lipid storage myopathy (Figure 3). Especially in adult cases, diagnosis is often not straight-forward, resulting in a significant diagnostic delay. One patient has been diagnosed as late as 29 years after the onset of symptoms. Surprisingly, the diagnostic delay was not less pronounced in patients with acute symptoms. For patients with acute decompensations early diagnosis and treatment are essential for survival and a positive outcome. Because inborn errors of metabolism are commonly thought to be “pediatric diseases”, awareness of these conditions among physicians treating adults is often low. Diagnostic work-up including MS/MS-based acylcarnitine profiling in dried blood spots/plasma and organic acid analysis in urine should be initiated promptly in every patient with acidosis of unknown origin. Additionally, MADD should be ruled out in any patient with suggestive muscular symptoms. As in patients with milder disease variants symptoms are often intermittent and only become evident during periods of illness and catabolic stress, late-onset MADD might be underdiagnosed [[29],[30]]. Organic acid and acylcarnitine analyses should be performed as first-line investigations with a low degree of suspicion as they may prevent patients from more invasive investigations like liver or muscle biopsies.

Making a biochemical diagnosis of late-onset MADD may be challenging in both symptomatic and asymptomatic individuals [[31]]. Most patients display a typical pattern of organic acids in urine and acylcarnitines in dried blood/plasma at least during metabolic decompensations. However, organic acid and acylcarnitine results may be unremarkable especially during metabolically stable conditions and in some cases even during catabolism [[7],[9],[13],[19],[31]-[36]]. Others show only selected abnormal metabolites, but not a full diagnostic pattern. Some therapeutic interventions like MCT oil, valproic acid or particular antibiotics (pivalic acid) can also mimic at least parts of the biochemical profile [[37],[38]]. Finally, due to common polymorphic alleles in the *ACADS* gene about 14% of the population will excrete ethylmalonic acid or will have elevated C4-acylcarnitine species in plasma [[39]]. This indicates, that MADD cannot be definitely excluded by a single metabolic screening test. The diagnosis should therefore rely on blood and urine analysis and ultimately be confirmed by molecular methods. In patients with muscular symptoms and histologically proven lipid storage myopathy but unremarkable metabolite patterns molecular analysis (starting with the *ETFDH* gene, followed by sequencing of *ETFA* and *ETFB*) is advisable.

Recently, several riboflavin transporters have been identified [[40]-[42]]: *SLC52A1* (RFT1), *SLC52A2* (RFT3) and *SLC52A3* (RFT2) are transmembrane proteins that mediate the cellular uptake of riboflavin [[42]]. Defects of these transporters lead to cellular riboflavin deficiency and may result in biochemical and clinical abnormalities mimicking MADD [[43],[44]]. Only one patient with a RFT1 defect has been identified so far [[43]]. This woman was clinically asymptomatic, but showed biochemical evidence of riboflavin deficiency. She was diagnosed after her daughter had presented with poor suck, hypoglycemia, and metabolic acidosis within the first days of life [[45]]. Other riboflavin transporter defects that can mimick mild MADD biochemically are the Brown-Vialetto-Van Laere (BVVL) and the Fazio Londe (FL) syndrome, two disorders which are nowadays considered to be the same disease entity [[44],[46],[47]]. These cases are due to mutations in the SLC52A2 or SLC52A3 genes which code for the RFT3 and RFT2 proteins, respectively. The clinical picture of these rare neurologic disorders usually differs from MADD and comprises sensorineural deafness (not in Fazio Londe syndrome), bulbar palsy, facial weakness and respiratory distress [[44],[47]]. However, a certain clinical overlap, especially with respect to the muscular symptoms, can make it difficult to distinguish late-onset MADD and BVVL/FL syndrome. It is well possible that some patients with genetic riboflavin transporter defects have been misdiagnosed with MADD. In particular, patients in whom no mutations in *ETFDH*, *ETFA* and *ETFB* can be identified should be investigated for a defective riboflavin transporter.

The positive clinical effects of riboflavin treatment are well documented as are the molecular mechanisms for riboflavin responsiveness [[4],[7],[8],[29],[48],[49]]. In particular, the chaperone effects that can compensate for inherited folding defects of ETFDH, have recently been described [[49]]. The vast majority of late-onset MADD patients respond very well to riboflavin. It has been shown that ETFDH deficiency may be associated with secondary coenzyme Q10 (Q10) deficiency. Thus, Q10 supplementation in addition to riboflavin treatment has been suggested [[50]]. The finding of *ETFDH* mutations in patients with the myopathic phenotype of Q10 deficiency initially lead to the suggestion that the late-onset form of MADD and the myopathic form of Q10 deficiency are allelic diseases [[50]]. However, in the meanwhile, late-onset MADD patients without Q10 deficiency have been reported contradicting this hypothesis [[16]]. Q10 deficiency has been found to be associated with increased mitochondrial production of reactive oxygen species (ROS) due to an electron leak of misfolded variant ETFDH proteins with impaired Q10 binding affinity [[49],[51]]. Based on studies with fibroblasts of six patients with riboflavin-responsive MADD Cornelius et al. have reported that Q10 treatment can decrease ROS production and may relieve oxidative stress. This suggests that late-onset MADD patients could benefit from a combined treatment of riboflavin and Q10 [[52]]. Q10 treatment alone is not advisable. Six Chinese patients, who were given Q10 alone before the diagnosis of late-onset MADD was made, showed only a mild response with their muscle strength never returning to normal [[28]]. Yamaguchi et al. studied the therapeutic effect of bezafibrate by an in vitro probe acylcarnitine assay using cultured fibroblasts of a patient with late-onset MADD [[53]]. The aberrantly elevated MADD-characteristic acylcarnitines were clearly corrected by the presence of bezafibrate in culture medium. Following these results, a clinical trial of bezafibrate was performed in the same patient, a 2-year-old boy, and resulted in a dramatical improve of his motor and cognitive skills in combination with a reduction of C4, C8, C10 and C12 acylcarnitines in blood and a normalization of the urinary organic acid profile. Notably, this boy has never been treated with riboflavin. Due to the loss of carnitine conjugates via the urine, MADD patients are prone to carnitine deficiency, which may require oral carnitine supplementation [[5],[9]]. Taken together, the mainstay of therapy in patients with late-onset MADD is riboflavin with excellent outcome data, and supplementation should be prompted immediately after diagnosis.

A clear genotype-phenotype correlation has been described for the different subtypes of MADD [[54]]: Homozygosity for null mutations is usually associated with MADD type 1, whereas even minor amounts of residual ETF/ETFDH activity seem to be sufficient to prevent embryonic development of congenital anomalies giving rise to type 2 disease. A relatively high residual activity is found in the late-onset form type 3 [[54]]. All patients with late-onset MADD of whom mutation data are published carry at least one missense variation, which may potentially result in some residual activity (Additional file 1: Table S1). The genotype-phenotype correlation within the group of late-onset patients, however, is poor. Apart from the disease-causing mutation other, especially exogenous, factors such as febrile infections, a restricted diet and other physiologic stressors may play an important role in the modulation of the clinical phenotype. Most mutations seem to be private. Three common mutations in the *ETFDH* gene have been described which are mainly found in the Chinese and Taiwanese population: c.250G > A (p.A84T), c.770A > G (p.Y257C), c.1227A > C (p.L409F) [[23],[28],[55]]. As this literature review has shown, the vast majority of late-onset patients harbour mutations in the *ETFDH* gene, while *ETFA* or *ETFB* mutations are found in only about 7% of individuals. Therefore, mutation analysis in this cohort should first focus on *ETFDH*.

While early-onset MADD is a disease with high mortality, the prognosis of late-onset MADD seems to be good. Nevertheless, 5% of patients reported in the literature had died mainly during metabolic decompensations. In several patients, death could not be prevented despite the known diagnosis of MADD. Although most deaths occurred in childhood, several cases with fatal outcome during adolescence and adulthood have been described [[22],[56]]. Even patients who present with chronic symptoms in adulthood are prone to metabolic crises. To prevent unfavorable outcomes, patients need to be aware of the disease risks and potential triggers of decompensations. MADD represents an official target of newborn screening in only few countries, e.g. in the United States. Limited data exist on the detection rate of late-onset MADD by this approach. However, based on the number of detected cases so far, it is quite possible that most MADD patients are recognized by newborn screening [[57]]. Early diagnosis and treatment from the neonatal period might modify the clinical outcome and possibly even prevent symptoms in late-onset MADD patients.

In conclusion, late-onset MADD may be an underdiagnosed disease in adults with myopathy and should be considered early in the differential diagnosis. This may not only prevent patients from invasive diagnostic procedures such as muscle biopsies, but also help to avoid fatal metabolic decompensations.

## Competing interests

The author has no competing interests to declare.

## Additional file

## Supplementary Material

Additional file 1: Table S1. Clinical, biochemical and molecular data of 350 patients with late-onset multiple acyl-CoA dehydrogenase deficiency published in the literature between 1979 and 2014. Patient numbers in this supplemental table do not correspond to those displayed in Figure 1. Click here for file

## References

[B1] Frerman FE Goodman SI Scriver CR, Sly WS, Childs B, Beaudet AL, Valle D, Kinzler KW, Vogelstein B Defects of Electron Transfer Flavoprotein and Electron Transfer Flavoprotein-Ubiquinone Oxidoreductase: Glutaric Acidemia Type II The Metabolic and Molecular Bases of Inherited Disease 2001

[B2] Goodman SI Frerman FE Glutaric acidaemia type II (multiple acyl-CoA dehydrogenation deficiency) J Inherit Metab Dis 1984 7 Suppl 1 33 37 6434842 10.1007/BF03047371

[B3] Dusheiko G Kew MC Joffe BI Lewin JR Mantagos S Tanaka K Recurrent hypoglycemia associated with glutaric aciduria type II in an adult N Engl J Med 1979 301 26 1405 1409 514320 10.1056/NEJM197912273012601

[B4] Bell RB Brownell AK Roe CR Engel AG Goodman SI Frerman FE Seccombe DW Snyder FF Electron transfer flavoprotein: ubiquinone oxidoreductase (ETF:QO) deficiency in an adult Neurology 1990 40 11 1779 1782 2234436 10.1212/wnl.40.11.1779

[B5] Izumi R Suzuki N Nagata M Hasegawa T Abe Y Saito Y Mochizuki H Tateyama M Aoki M A case of late onset riboflavin-responsive multiple acyl-CoA dehydrogenase deficiency manifesting as recurrent rhabdomyolysis and acute renal failure Intern Med 2011 50 21 2663 2668 22041377 10.2169/internalmedicine.50.5172

[B6] Sugai F Baba K Toyooka K Liang WC Nishino I Yamadera M Sumi H Fujimura H Nishikawa Y Adult-onset multiple acyl CoA dehydrogenation deficiency associated with an abnormal isoenzyme pattern of serum lactate dehydrogenase Neuromuscul Disord 2012 22 2 159 161 21907580 10.1016/j.nmd.2011.08.004

[B7] Wasant P Kuptanon C Vattanavicharn N Liammongkolkul S Ratanarak P Sangruchi T Yamaguchi S Glutaric aciduria type 2, late onset type in Thai siblings with myopathy Pediatr Neurol 2010 43 4 279 282 20837308 10.1016/j.pediatrneurol.2010.05.018

[B8] Ishii K Komaki H Ohkuma A Nishino I Nonaka I Sasaki M Central nervous system and muscle involvement in an adolescent patient with riboflavin-responsive multiple acyl-CoA dehydrogenase deficiency Brain Dev 2010 32 8 669 672 19783111 10.1016/j.braindev.2009.08.008

[B9] Mareska MC Adams KK Muenzer J Frerman F Braun TG Howard JF Jr Adult-onset presentation of Glutaric Acidemia Type II with Myopathy J Clin Neuromuscul Dis 2003 4 3 124 128 19078703 10.1097/00131402-200303000-00005

[B10] Morris AAM Spiekerkoetter U Saudubray J-M, Berghe G, Walter JH Disorders of Mitochondrial Fatty Acid Oxidation and Related Metabolic Pathways Inborn Metabolic Diseases, Diagnosis and Treatment 2012 Springer-Verlag Berlin Heidelberg New York, New York 201 212

[B11] Amendt BA Rhead WJ The multiple acyl-coenzyme A dehydrogenation disorders, glutaric aciduria type II and ethylmalonic-adipic aciduria. Mitochondrial fatty acid oxidation, acyl-coenzyme A dehydrogenase, and electron transfer flavoprotein activities in fibroblasts J Clin Invest 1986 78 1 205 213 3722376 10.1172/JCI112553 PMC329551

[B12] Harpey JP Charpentier C Coude M Divry P Paturneau-Jouas M Sudden infant death syndrome and multiple acyl-coenzyme A dehydrogenase deficiency, ethylmalonic-adipic aciduria, or systemic carnitine deficiency J Pediatr 1987 110 6 881 884 3585604 10.1016/s0022-3476(87)80401-8

[B13] Russell AP Schrauwen P Somm E Gastaldi G Hesselink MK Schaart G Kornips E Lo SK Bufano D Giacobino JP Muzzin P Ceccon M Angelini C Vergani L Decreased fatty acid beta-oxidation in riboflavin-responsive, multiple acylcoenzyme A dehydrogenase-deficient patients is associated with an increase in uncoupling protein-3 J Clin Endocrinol Metab 2003 88 12 5921 5926 14671191 10.1210/jc.2003-030885

[B14] Grice AS Peck TE Multiple acyl-CoA dehydrogenase deficiency: a rare cause of acidosis with an increased anion gap Br J Anaesth 2001 86 3 437 441 11573539 10.1093/bja/86.3.437

[B15] Vergani L Barile M Angelini C Burlina AB Nijtmans L Freda MP Brizio C Zerbetto E Dabbeni-Sala F Riboflavin therapy. Biochemical heterogeneity in two adult lipid storage myopathies Brain 1999 122 Pt 12 2401 2411 10581232 10.1093/brain/122.12.2401

[B16] Liang WC Ohkuma A Hayashi YK Lopez LC Hirano M Nonaka I Noguchi S Chen LH Jong YJ Nishino I ETFDH mutations, CoQ10 levels, and respiratory chain activities in patients with riboflavin-responsive multiple acyl-CoA dehydrogenase deficiency Neuromuscul Disord 2009 19 3 212 216 19249206 10.1016/j.nmd.2009.01.008 PMC10409523

[B17] Burns SP Holmes HC Chalmers RA Johnson A Iles RA Proton NMR spectroscopic analysis of multiple acyl-CoA dehydrogenase deficiency–capacity of the choline oxidation pathway for methylation in vivo Biochim Biophys Acta 1998 1406 3 274 282 9630673 10.1016/s0925-4439(98)00015-5

[B18] Cotelli MS Vielmi V Rimoldi M Rizzetto M Castellotti B Bertasi V Todeschini A Gregorelli V Baronchelli C Gellera C Padovani A Filosto M Riboflavin-responsive multiple acyl-CoA dehydrogenase deficiency with unknown genetic defect Neurol Sci 2012 33 6 1383 1387 22190129 10.1007/s10072-011-0900-1

[B19] Wen B Dai T Li W Zhao Y Liu S Zhang C Li H Wu J Li D Yan C Riboflavin-responsive lipid-storage myopathy caused by ETFDH gene mutations J Neurol Neurosurg Psychiatry 2010 81 2 231 236 19758981 10.1136/jnnp.2009.176404

[B20] Law LK Tang NL Hui J Fung SL Ruiter J Wanders RJ Fok TF Lam CW Novel mutations in ETFDH gene in Chinese patients with riboflavin-responsive multiple acyl-CoA dehydrogenase deficiency Clin Chim Acta 2009 404 2 95 99 19265687 10.1016/j.cca.2009.02.015

[B21] Schiff M Froissart R Olsen RK Acquaviva C Vianey-Saban C Electron transfer flavoprotein deficiency: functional and molecular aspects Mol Genet Metab 2006 88 2 153 158 16510302 10.1016/j.ymgme.2006.01.009

[B22] Fitzgerald M Crushell E Hickey C Cyclic vomiting syndrome masking a fatal metabolic disease Eur J Pediatr 2013 172 5 707 710 23052622 10.1007/s00431-012-1852-z

[B23] Lan MY Fu MH Liu YF Huang CC Chang YY Liu JS Peng CH Chen SS High frequency of ETFDH c.250G > A mutation in Taiwanese patients with late-onset lipid storage myopathy Clin Genet 2010 78 6 565 569 20370797 10.1111/j.1399-0004.2010.01421.x

[B24] Triggs WJ Roe CR Rhead WJ Hanson SK Lin SN Willmore LJ Neuropsychiatric manifestations of defect in mitochondrial beta oxidation response to riboflavin J Neurol Neurosurg Psychiatry 1992 55 3 209 211 1564483 10.1136/jnnp.55.3.209 PMC1014728

[B25] Scheicht D Werthmann ML Zeglam S Holtmeier J Holtmeier W Strunk J [Muscle weakness and early stages of liver failure in a 22-year-old man] Internist (Berl) 2013 54 8 1016 1022 23900454 10.1007/s00108-013-3329-1

[B26] Olsen RK Olpin SE Andresen BS Miedzybrodzka ZH Pourfarzam M Merinero B Frerman FE Beresford MW Dean JC Cornelius N Andersen O Oldfors A Holme E Gregersen N Turnbull DM Morris AA ETFDH mutations as a major cause of riboflavin-responsive multiple acyl-CoA dehydrogenation deficiency Brain 2007 130 Pt 8 2045 2054 17584774 10.1093/brain/awm135

[B27] Papadimitriou A Servidei S Late onset lipid storage myopathy due to multiple acyl CoA dehydrogenase deficiency triggered by valproate Neuromuscul Disord 1991 1 4 247 252 1822802 10.1016/0960-8966(91)90097-c

[B28] Xi J Wen B Lin J Zhu W Luo S Zhao C Li D Lin P Lu J Yan C Clinical features and ETFDH mutation spectrum in a cohort of 90 Chinese patients with late-onset multiple acyl-CoA dehydrogenase deficiency J Inherit Metab Dis 2013 37 399 404 24357026 10.1007/s10545-013-9671-6

[B29] Henriques BJ Rodrigues JV Olsen RK Bross P Gomes CM Role of flavinylation in a mild variant of multiple acyl-CoA dehydrogenation deficiency: a molecular rationale for the effects of riboflavin supplementation J Biol Chem 2009 284 7 4222 4229 19088074 10.1074/jbc.M805719200

[B30] Koppel S Gottschalk J Hoffmann GF Waterham HR Blobel H Kolker S Late-onset multiple acyl-CoA dehydrogenase deficiency: a frequently missed diagnosis? Neurology 2006 67 8 1519 17060596 10.1212/01.wnl.0000240065.35635.a6

[B31] Pollard LM Williams NR Espinoza L Wood TC Spector EB Schroer RJ Holden KR Diagnosis, treatment, and long-term outcomes of late-onset (type III) multiple acyl-CoA dehydrogenase deficiency J Child Neurol 2010 25 8 954 960 20023066 10.1177/0883073809351984

[B32] Kaminsky P Acquaviva-Bourdain C Jonas J Pruna L Chaloub GE Rigal O Grignon Y Vianey-Saban C Subacute myopathy in a mature patient due to multiple acyl-coenzyme A dehydrogenase deficiency Muscle Nerve 2011 43 3 444 446 21321959 10.1002/mus.21881

[B33] Mumtaz HA Gupta V Singh P Marwaha RK Khandelwal N MR imaging findings of glutaric aciduria type II Singapore Med J 2010 51 4 e69 71 20505899

[B34] Takken T Custers J Visser G Dorland L Helders P de Koning T Prolonged exercise testing in two children with a mild Multiple Acyl-CoA-Dehydrogenase deficiency Nutr Metab (Lond) 2005 2 1 12 15907213 10.1186/1743-7075-2-12 PMC1159171

[B35] Gianazza E Vergani L Wait R Brizio C Brambilla D Begum S Giancaspero TA Conserva F Eberini I Bufano D Angelini C Pegoraro E Tramontano A Barile M Coordinated and reversible reduction of enzymes involved in terminal oxidative metabolism in skeletal muscle mitochondria from a riboflavin-responsive, multiple acyl-CoA dehydrogenase deficiency patient Electrophoresis 2006 27 5–6 1182 1198 16470778 10.1002/elps.200500687

[B36] Van Hove JL Grunewald S Jaeken J Demaerel P Declercq PE Bourdoux P Niezen-Koning K Deanfeld JE Leonard JV D, L-3-hydroxybutyrate treatment of multiple acyl-CoA dehydrogenase deficiency (MADD) Lancet 2003 361 9367 1433 1435 12727399 10.1016/S0140-6736(03)13105-4

[B37] Boemer F Schoos R de Halleux V Kalenga M Debray FG Surprising causes of C5-carnitine false positive results in newborn screening Mol Genet Metab 2014 111 1 52 54 24291264 10.1016/j.ymgme.2013.11.005

[B38] Abdenur JE Chamoles NA Guinle AE Schenone AB Fuertes AN Diagnosis of isovaleric acidaemia by tandem mass spectrometry: false positive result due to pivaloylcarnitine in a newborn screening programme J Inherit Metab Dis 1998 21 6 624 630 9762597 10.1023/a:1005424331822

[B39] Corydon MJ Vockley J Rinaldo P Rhead WJ Kjeldsen M Winter V Riggs C Babovic-Vuksanovic D Smeitink J De Jong J Levy H Sewell AC Roe C Matern D Dasouki M Gregersen N Role of common gene variations in the molecular pathogenesis of short-chain acyl-CoA dehydrogenase deficiency Pediatr Res 2001 49 1 18 23 11134486 10.1203/00006450-200101000-00008

[B40] Yonezawa A Masuda S Katsura T Inui K Identification and functional characterization of a novel human and rat riboflavin transporter, RFT1 Am J Physiol Cell Physiol 2008 295 3 C632 641 18632736 10.1152/ajpcell.00019.2008

[B41] Yamamoto S Inoue K Ohta KY Fukatsu R Maeda JY Yoshida Y Yuasa H Identification and functional characterization of rat riboflavin transporter 2 J Biochem 2009 145 4 437 443 19122205 10.1093/jb/mvn181

[B42] Yao Y Yonezawa A Yoshimatsu H Masuda S Katsura T Inui K Identification and comparative functional characterization of a new human riboflavin transporter hRFT3 expressed in the brain J Nutr 2010 140 7 1220 1226 20463145 10.3945/jn.110.122911

[B43] Ho G Yonezawa A Masuda S Inui K Sim KG Carpenter K Olsen RK Mitchell JJ Rhead WJ Peters G Christodoulou J Maternal riboflavin deficiency, resulting in transient neonatal-onset glutaric aciduria Type 2, is caused by a microdeletion in the riboflavin transporter gene GPR172B Hum Mutat 2011 32 1 E1976 1984 21089064 10.1002/humu.21399

[B44] Bosch AM Abeling NG Ijlst L Knoester H van der Pol WL Stroomer AE Wanders RJ Visser G Wijburg FA Duran M Waterham HR Brown-Vialetto-Van Laere and Fazio Londe syndrome is associated with a riboflavin transporter defect mimicking mild MADD: a new inborn error of metabolism with potential treatment J Inherit Metab Dis 2011 34 1 159 164 21110228 10.1007/s10545-010-9242-z PMC3026695

[B45] Chiong MA Sim KG Carpenter K Rhead W Ho G Olsen RK Christodoulou J Transient multiple acyl-CoA dehydrogenation deficiency in a newborn female caused by maternal riboflavin deficiency Mol Genet Metab 2007 92 1–2 109 114 17689999 10.1016/j.ymgme.2007.06.017

[B46] Dipti S Childs AM Livingston JH Aggarwal AK Miller M Williams C Crow YJ Brown-Vialetto-Van Laere syndrome; variability in age at onset and disease progression highlighting the phenotypic overlap with Fazio-Londe disease Brain Dev 2005 27 6 443 446 16122634 10.1016/j.braindev.2004.10.003

[B47] Bosch AM Stroek K Abeling NG Waterham HR Ijlst L Wanders RJ The Brown-Vialetto-Van Laere and Fazio Londe syndrome revisited: natural history, genetics, treatment and future perspectives Orphanet J Rare Dis 2012 7 83 23107375 10.1186/1750-1172-7-83 PMC3517535

[B48] Rosa M Pascarella A Parenti G Buono S Romano A Della Casa R Andria G Marino M Riccio MP Bravaccio C Developmental evolution in a patient with multiple acyl-coenzymeA dehydrogenase deficiency under pharmacological treatment Eur J Paediatr Neurol 2012 16 2 203 205 21868270 10.1016/j.ejpn.2011.07.003

[B49] Cornelius N Frerman FE Corydon TJ Palmfeldt J Bross P Gregersen N Olsen RK Molecular mechanisms of riboflavin responsiveness in patients with ETF-QO variations and multiple acyl-CoA dehydrogenation deficiency Hum Mol Genet 2012 21 15 3435 3448 22611163 10.1093/hmg/dds175

[B50] Gempel K Topaloglu H Talim B Schneiderat P Schoser BG Hans VH Palmafy B Kale G Tokatli A Quinzii C Hirano M Naini A DiMauro S Prokisch H Lochmüller H Horvath R The myopathic form of coenzyme Q10 deficiency is caused by mutations in the electron-transferring-flavoprotein dehydrogenase (ETFDH) gene Brain 2007 130 Pt 8 2037 2044 17412732 10.1093/brain/awm054 PMC4345103

[B51] Cornelius N Byron C Hargreaves I Guerra PF Furdek AK Land J Radford WW Frerman F Corydon TJ Gregersen N Olsen RK Secondary coenzyme Q10 deficiency and oxidative stress in cultured fibroblasts from patients with riboflavin responsive multiple Acyl-CoA dehydrogenation deficiency Hum Mol Genet 2013 22 19 3819 3827 23727839 10.1093/hmg/ddt232

[B52] Cornelius N Corydon TJ Gregersen N Olsen RK Cellular consequences of oxidative stress in riboflavin responsive multiple acyl-CoA dehydrogenation deficiency patient fibroblasts Hum Mol Genet 2014 ᅟ ᅟ Epub ahead of print 10.1093/hmg/ddu146 24698980

[B53] Yamaguchi S Li H Purevsuren J Yamada K Furui M Takahashi T Mushimoto Y Kobayashi H Hasegawa Y Taketani T Fukao T Fukuda S Bezafibrate can be a new treatment option for mitochondrial fatty acid oxidation disorders: evaluation by in vitro probe acylcarnitine assay Mol Genet Metab 2012 107 1–2 87 91 22841441 10.1016/j.ymgme.2012.07.004

[B54] Olsen RK Andresen BS Christensen E Bross P Skovby F Gregersen N Clear relationship between ETF/ETFDH genotype and phenotype in patients with multiple acyl-CoA dehydrogenation deficiency Hum Mutat 2003 22 1 12 23 12815589 10.1002/humu.10226

[B55] Wang ZQ Chen XJ Murong SX Wang N Wu ZY Molecular analysis of 51 unrelated pedigrees with late-onset multiple acyl-CoA dehydrogenation deficiency (MADD) in southern China confirmed the most common ETFDH mutation and high carrier frequency of c.250G>A J Mol Med (Berl) 2011 89 6 569 576 21347544 10.1007/s00109-011-0725-7

[B56] Lee HC Lai CK Siu TS Yuen YP Chan KY Chan AY Tam S Mak CM Lam CW Role of postmortem genetic testing demonstrated in a case of glutaric aciduria type II Diagn Mol Pathol 2010 19 3 184 186 20736750 10.1097/PDM.0b013e3181c9a8a8

[B57] McHugh D Cameron CA Abdenur JE Abdulrahman M Adair O Al Nuaimi SA Ahlman H Allen JJ Antonozzi I Archer S Au S Auray-Blais C Baker M Bamforth F Beckmann K Pino GB Berberich SL Binard R Boemer F Bonham J Breen NN Bryant SC Caggana M Caldwell SG Camilot M Campbell C Carducci C Bryant SC Caggana M Caldwell SG Clinical validation of cutoff target ranges in newborn screening of metabolic disorders by tandem mass spectrometry: a worldwide collaborative project Genet Med 2011 13 3 230 254 21325949 10.1097/GIM.0b013e31820d5e67

