# Supplementary material for: Clinical and genetical heterogeneity of late-onset multiple acyl-coenzyme A dehydrogenase deficiency
Source: Orphanet J Rare Dis. 2014 Jul 22;9:117. doi: 10.1186/s13023-014-0117-5 (PMC4222585; doi:10.1186/s13023-014-0117-5)
Supplement: Additional file 1: Table S1. — Clinical, biochemical and molecular data of 350 patients with late-onset multiple acyl-CoA dehydrogenase deficiency published in the literature between 1979 and 2014. Patient numbers in this supplemental table do not correspond to those displayed in Figure 1. [file s13023-014-0117-5-S1.doc]

Additional file 1: Table S1

Clinical, biochemical and genetic data of 350 late-onset MADD patients reported in the literature

| # | Sex | Age at onset | Age at diagnosis | Presentation | | | Typical abnormalities in the metabolite profiles | | Response to riboflavine therapy | Genotype | | | Author |
| --- | --- | --- | --- | --- | --- | --- | --- | --- | --- | --- | --- | --- | --- |
|  |  |  |  | acute | chronic | clinical features | OA | AC |  | affected gene | Nucleotide change  allele 1  allele 2 | Amino acid change  (predicted from c.DNA) |  |
| 1 | f | 6 mo | 20 y | X | - | episodic vomiting during childhood, acute metabolic decompensation with cardiovascular collapse and multiorgan failure, deceased at age 20 years | n.a. | n.a. | n.a. | ETFDH | c.51_52ins c.1367>T | p.A18Cfs*5  p.P456L | Fitzgerald 2013 [^1^](#_ENREF_1) |
| 2 | f | 4 y | 4 y | X | - | encephalopathy, hypoketotic hypoglycemia, acidosis | X | X | n.a. | ETFDH | c.1601C>T  c.1601C>T | p.P534L  p.P534L | Trakadis 2012 [^2^](#_ENREF_2) |
| 3 | f | 21 y | 21 y | - | X | increased fat deposition, especially in the  extremities, joint hyperflexibility, chronic fatigue and episodes of myalgia with increased CK related to stress | X | X | n.a. | ETFDH | c.1601C>T  c.1601C>T | p.P534L  p.P534L | Trakadis 2012 [^2^](#_ENREF_2) |
| 4 | m | n.a. | 62 y | X | X | excessive fatigability on exertion,  multiple acute hypoglycemic  decompensations | n.a. | n.a. | n.a. | ETFDH | c.1601C>T  c.1601C>T | p.P534L  p.P534L | Trakadis 2012 [^2^](#_ENREF_2) |
| 5 | m | ca. 45 y | 55 y | - | X | osphyalgia, dorsalgia, muscle weakness, anorexia and intermittent nausea, disgust with meat | X | X | X | ETFDH | c.1522C>A  c.1583_1584insA | p.P508T  p.N528Kfs*3 | Zhao 2012 [^3^](#_ENREF_3) |
| 6 | m | 46 y | 56 y | X | X | fatigability and weakness  in neck and lower limbs, 5 episodes of rhabdomyolysis followed  by acute renal failure and lactic acidosis, 1 episode with difficulty in breathing and severe muscle weakness  after fasting for a whole day | X | X | X | ETFDH | c.1211T>C  c.1786G>A | p.M404T  p.D596N | Izumi 2011 [^4^](#_ENREF_4) |
| 7 | f | 9 y  (1 y ?) | 11 y | X | X | hypotonia, elevated CK levels, hyperammonemia after a fever episode | X | X | n.a. | ETFDH | c.250G > A c.383T > C | p.A84T  p.F128S | Er 2011 [^5^](#_ENREF_5)  Chien 2014 [^6^](#_ENREF_6) |
| 8 | m | 36 y | 37 y | - | X | exercise intolerance in hip and thigh muscles | X | n.a. | X | ETFDH | c.1531G>A c.1809G>A | p.D511N  p.W603* | Sugai 2012 [^7^](#_ENREF_7) |
| 9 | m | 4 m | < 8 m | X | X | musular weakness, metabolic  acidosis and hypoglycemia | X | X | X | n.a. | n.a. | n.a. | Rosa 2012 [^8^](#_ENREF_8) |
| 10 | m | 41 y | 41 y | - | X | general weakness, at the age of 16 y episode of general weakness and elevated liver enzymes with  symptoms disappearing after treatment with multi-vitamins | n.a. | X | X | ETFDH | c.1218 G>C  c.1698 C>T | p.G296R  p.P456P | Lämmer 2011 [^9^](#_ENREF_9) |
| 11-66 | 24 f, 32 m | mean 24.5±12.6 y  (4-55 y) | n.a. | - | X | progressive and/or fluctuating myopathy, mainly affecting the proximal limb muscles and particularly the neck flexors and extensors, exercise intolerance, extreme fatigue, myalgia in 29/56, dysphagia in 12/56, tachydyspnea in 6/56  cyclic vomiting in 17/56,  deterioration of symptoms  upon infection or metabolic stress, no acute decompensations with hypoglycemia, acidosis or encephalopathy | X* | X | X | ETFDH | 39 patients:  c.250G>A  c.250G>A  6 patients:  c.250G>A  c.524G>A  2 patients  c.250G>A  c.643G>A  2 patients:  c.250G>A  c.770A>G  1 patient:  c.250G>A c.998A>G  1 patient:  c.524G>A c.770A>G  1 patient:  c.770A>G  c.1254-1257del  1 patient:  c.1395T>G  c. ? | p.A84T  p.A84T  p.A84T  p.R175H  p.A84T  p.A215T  p.A84T  p.Y257C  p.A84T  p.Y333C  p.R175H  p.Y257C  p.Y257C  p.L418Tfs*10  p.Y465*  p. ? | Wang 2011 [^10^](#_ENREF_10) |
| 67 | f | 55 y | 55 y | - | X | myalgia and muscle weakness | - | X | X | ETFDH | c.877C>G  c.? | p.H293D  p.? | Kaminsky 2011 [^11^](#_ENREF_11) |
| 68 | m | 4 mo | 4 y | X | X | severe hypoglycemia and lethargy during infection, recurrent vomiting  associated with mild hyperammonemia, dehydration requiring frequent hospitalizations, generalized weakness | X | X^§^ | X | ETFDH | c.820G>T  c.1601C>T | p.G274*  p.P534L | Wolfe 2010 [^12^](#_ENREF_12) |
| 69 | m | 9 y | 9 y | - | X | proximal muscle weakness | - | X | X | ETFDH | c.250G>A  c.832-1G>A | p.A84T  exon 8 skipping | Wasant 2010 [^13^](#_ENREF_13) |
| 70 | m | 7 y | 7 y | - | X | proximal muscle weakness after physical exercise | X | X | X | ETFDH | c.250G>A  c.832-1G>A | p.A84T  exon 8 skipping | Wasant 2010 [^13^](#_ENREF_13) |
| 71 | m | 14 y | postmortem | X | - | episode of self-limited vomiting at age 6 years, normal development, at age 14 years severe metabolic decompensation with hypoglycaemia, acidosis, hyperammonemia, respiratory distress and coma, multiple episodes of cardiac arrest, deceased, twin sister had died  of unknown cause in infancy | X | X | n.a. | ETFA | c.502G>T  c.786A>G | p.V168F  p.Q262R | Lee 2010 [^14^](#_ENREF_14) |
| 72 | m | 7 mo | 7 mo | X | - | « peculiar sweat sock breath odour”, altered sensorium, hypotonia, hypoglycemia, elevated anion gap | - | n.a. | n.a. | n.a. | n.a. | n.a. | Mumtaz 2010 [^15^](#_ENREF_15) |
| 73 | m | 42 y | 56 y | - | X | progressive proximal myopathy, intermittent weakness and severe muscle pain predominantly of the proximal limbs after physical exercise, profuse sweating | X | X | X | ETFDH | c.728T>C  c.881C>T | p.I243T  p.T294I | Köppel 2006 [^16^](#_ENREF_16) |
| 74 | m | 3.1 y | 3.1 y | X | - | coma, hypoglycemia | -/X^#^ | -/X^$#^ | n.a. | n.a. | n.a. | n.a. | Takken 2005 [^17^](#_ENREF_17) |
| 75 | m | - | ca. 5 y | - | - | asymptomatic | - | -^$^ | n.a. | n.a. | n.a. | n.a. | Takken 2005 [^17^](#_ENREF_17) |
| 76 | f | 6 mo | n.a. | - | X | hypotonia and hepatomegaly at age 6 months, delayed motor development, lipid storage myopathy, persisting hepatomegaly and mild weakness under therapy, at age 14 years marked hypoventilation (nonobstructive), intermittend ventilatory assistance, brother had died at 3 months with hepatic steatosis | X | X | (X) | ETFDH | c.413T>G  c.IVS3+3A>T | p.L138R  truncated transcript | Olsen 2004 [^18^](#_ENREF_18)/ Turnbull 1988 [^19^](#_ENREF_19) |
| 77 | n.a | n.a. | n.a. | - | X | lipid storage myopathy | X | n.a. | X | n.a. | n.a. | n.a. | Liang 2003 [^20^](#_ENREF_20) |
| 78 | n.a. | n.a. | n.a. | X | X | intermittent episodes of non-ketotic hypoglycemia and muscle weakness | X | n.a. | X | n.a. | n.a. | n.a. | Liang 2003 [^20^](#_ENREF_20) |
| 79 | f | 31 y | 31 y | X | - | acute decompensation with vomiting and confusion, hypoglycaemia, acidosis, severe circulatory failure, oliguria | X | n.a. | limited efficacy | n.a. | n.a. | n.a. | Grice 2001 [^21^](#_ENREF_21) |
| 80 | m | 6 mo | 6 mo | n.a. | X | severe muscle weakness, lipid storage myopathy and fatty liver, normal development | X | n.a. | X | n.a. | n.a. | n.a. | Tojo 2000 [^22^](#_ENREF_22) |
| 81 | m | 36 y | 36 y | - | X | progressive generalized muscle weakness, tetraplegia | -^&^ | X | X | n.a. | n.a. | n.a. | Vergani 1999 [^23^](#_ENREF_23)/ Mareska 2003 [^24^](#_ENREF_24) |
| 82 | m | 25 y | 25 y | - | X | depressive episode associated with loss od appetite, heavy drinking, vomiting and nausea, concomitant weight loss of 11 kg, muscle pain and fatigue | X^&^ | X^&^ | limited efficacy | n.a. | n.a. | n.a. | Vergani 1999 [^23^](#_ENREF_23)/ Mareska 2003 [^24^](#_ENREF_24) |
| 83 | m | 3 y | 3 y | - | X | tremor of the upper limbs, paraparetic ataxic gait and slurred speech, loss of the ability to walk alone and to speak | X | X | X | n.a. | n.a. | n.a. | Uziel 1995 [^25^](#_ENREF_25) |
| 84 | m. | 6 w | 6 w | X | X | hypoketotic hypoglycemia | X | n.a. | X | n.a. | n.a. | n.a. | Onkenhout 1995 [^26^](#_ENREF_26)/ Mooy 1984 [^27^](#_ENREF_27) |
| 85 | n.a. | n.a. | n.a. | X | n.a. | hypoketotic hypoglycemia | X | n.a | n.a. | n.a. | n.a. | n.a. | Onkenhout 1995 [^26^](#_ENREF_26)/ Manning 1990 [^28^](#_ENREF_28) |
| 86 | f | 68 y | 69 y | X | X | muscle weakness, easy fatigability, muscle wasting, lipid storage myopathy, occasionally low blood glucose | X^&^ | n.a. | X | n.a. | n.a. | n.a. | Antozzi 1994 [^29^](#_ENREF_29)/ Mareska 2003 [^24^](#_ENREF_24) |
| 87 | m | 50 y | 62 y | - | X | several episodes of fatigability of lower limbs (at ages 50, 52, 53, and 57 years), normal psychomotor development, lipid storage myopathy | X^&^ | n.a. | X | n.a. | n.a. | n.a. | Araki 1994 [^30^](#_ENREF_30)/ Mareska 2003 [^24^](#_ENREF_24) |
| 88 | f | 29 y | 29 y | X | X | valproate-induced coma, complex partial seizures, behavioural disturbances, vomiting, severe headache, episodes of vomiting and altered behaviour, suspected dysthymic disorder and a mixed personalilty disorder with borderline and histrionic character traits | X^&^ | n.a. | X | n.a. | n.a. | n.a. | Triggs 1992 [^31^](#_ENREF_31) |
| 89 | f | 10 y | 25 y | X | X | episodes of muscle weakness, nausea and vomiting since the age of 10 | X | n.a. | n.a. | n.a. | n.a. | n.a. | Mongini 1992 [^32^](#_ENREF_32) |
| 90 | m | 18 y | 47 y | - | X | four major seizures at age 18 years, at age 43 years, nocturnal calf cramps and exercise intolerance, muscle pain on physical exercise, shortness of breath, difficulty in chewing | n.a. | n.a. | X | n.a. | n.a. | n.a. | Papadimitriou 1991 [^33^](#_ENREF_33) |
| 91 | f | 9 y | 12 y | X | X | generalized muscle weakness and easy fatigability, at 11 years  poor general condition, hepatomegaly and severe generalized muscle weakness and wasting, blood glucose  occasionally low, brother  died at age 8 years of an acute illness characterized by mental  confusion, hypotonia, muscle weakness, and hepatomegaly | X | n.a. | X | n.a. | n.a. | n.a. | Di Donato 1989 [^34^](#_ENREF_34)/ Peluchetti 1991 [^35^](#_ENREF_35)/ Mareska 2003 [^24^](#_ENREF_24) |
| 92 | f | 7 w | 4.5 y | X | - | brief episode  of tonic seizures (3-4 s) at age 7 weeks, 2 episodes of vomiting,  lethargy, coma and convulsions, acidosis, and hypoglycaemia in childhood | X | n.a. | n.a. | n.a. | n.a. | n.a. | Amendt 1986 [^36^](#_ENREF_36)/ Mantagos 1979 [^37^](#_ENREF_37) |
| 93 | m | early infancy | early infancy | - | X | Hyperexcitability and feeding difficulties in the neonatal period, normal development with disappearance of clinical abnormalities after the age of 6 months | X | n.a. | n.a. | n.a. | n.a. | n.a. | Amendt 1986 [^36^](#_ENREF_36)/ Duran 1983 [^38^](#_ENREF_38) |
| 94 | m | early, < 22 mo | 2.8 y | X | X | developmental delay, at age 22 months febrile illness followed by gait difficulty and irritability for 1 week, 2 episodes of febrile illnesses followed by 12-h periods of shaking and fine tremors at age 2 years, at age 34 months seizures and coma, mild acidosis, since then neurologically devastated | X | n.a. | n.a. | n.a. | n.a. | n.a. | Amendt 1986 [^36^](#_ENREF_36)/ Rhead 1987 [^39^](#_ENREF_39) |
| 95 | m | < 6 mo | 1.5 y | - | X | slow physical and mental development, failure to thrive | X○ | n.a. | n.a. | n.a. | n.a. | n.a. | Amendt 1986 [^36^](#_ENREF_36)/ Rhead 1987 [^39^](#_ENREF_39) |
| 96 | f | adulthood | n.a. | n.a. | n.a. | n.a. | n.a. | n.a. | n.a. | n.a. | n.a. | n.a. | Amendt 1986 [^36^](#_ENREF_36) |
| 97 | m | 5 y | 5 y | - | X | presentation with medulloblastoma, mental retardation, diagnosis due to abnormal brain MRI | X | n.a. | n.a. | n.a. | n.a. | n.a. | Yazici 2009 [^40^](#_ENREF_40) |
| 98 | f | 3 y | 7 y | - | X | muscle weakness in childhood, at age 14 years after stop of riboflavin therapy poor appetite, weight loss, lethargy and proximal muscle weakness | X | n.a. | X | ETFDH | c.1355delG  c.250G>A | p.R452Kfs*3  p.A84T | Law 2009 [^41^](#_ENREF_41) |
| 99 | f | 10 mo | 4 y | X | X | generalized floppiness following a chest infection at age 10 months, otherwise normal development, at age 4 years hypoglycemic convulsion, generalized hypotonia and mild hepatomegaly | X | n.a. | X | ETFDH | c.409CNT  c.1400GNC | p.P137S  p.G467R | Law 2009 [^41^](#_ENREF_41) |
| 100 | f | 18 mo | 18 mo | - | - | Sister of # 99, diagnosed following presentation of elder sister  still asymptomatic at age 14 years | X | n.a. | X | ETFDH | c.409CNT  c.1400GNC | p.P137S  p.G467R | Law 2009 [^41^](#_ENREF_41) |
| 101 | m | 2 y | 2 y | X | - | severe disease symptoms in association with a viral  infection and fever, deceased | n.a. | n.a. | n.a. | ETFB | c.382G>A  c.382G>A | p.D128N  p.D128N | Olsen 2003 [^42^](#_ENREF_42)/ Henriques 2009 [^43^](#_ENREF_43) |
| 102 | m | 5 mo | NBS | X | X | failure to thrive, slight developmental delay, at age 6 months acute presentation with acidosis, hepatomegaly, decreased cardiac function, severe hypertrophic cardiomyopathy, pericardial effusion, death due to ventricular  fibrillation, followed by cardiac arrest | n.a. | X | n.a. | n.a. | n.a. | n.a. | Singla 2008 [^44^](#_ENREF_44) |
| 103 | f | 21 mo | postmortem | X | - | found dead in bed after two days of illness with varicella and fever | X | n.a. | n.a. | n.a. | n.a. | n.a. | Lundemose 1997 [^45^](#_ENREF_45)/ Olsen 2003 [^42^](#_ENREF_42) |
| 104 | f | 15 y | 15 y | X | - | Reye-like episode with coma, hypoglycaemia, hyperammonemia and ketonuria, CK-MB 1310 U/l , peculiar ECG-abnormalities compatible with a subendocardic ischemic lesion | X | X | n.a. | n.a. | n.a. | n.a. | Benigno 2002 [^46^](#_ENREF_46) |
| 105 | m | 12 y | 20 y | - | X | difficulty in walking and climbing stairs since age 12 years, thigh and lumbar pain, decline in mood and cognitive performances at age 18 years | X | X | X | no mutations in ETFDH, ETFA and ETFB detectable | n.a. | n.a. | Cotelli 2012 [^47^](#_ENREF_47) |
| 106 | m | 5 mo | n.a. | X | X | Hypotonia and hypoglycemia, normal development | X | n.a. | X | ETFDH | c.922T>G  c.? | p.F308V  p.? | Yotsumoto 2008 [^48^](#_ENREF_48) |
| 107 | m | 6 mo | n.a. | - | X | hypotonia, normal development | X | n.a. | X | ETFDH | c.1208C>T  c.1208C>T | p.A403V  p.A403V | Yotsumoto 2008 [^48^](#_ENREF_48) |
| 108 | m | 8 mo | n.a. | n.a. | n.a. | Poor feeding, CPK elevation, and cardiomyopathy, deceased | X | n.a. | n.a. | ETFA | c.283T>G  c.283T>G | p.L95V  p.L95V | Yotsumoto 2008 [^48^](#_ENREF_48) |
| 109 | m | 16 mo | n.a. | X | X | Hypotonia, hypoglycemia, and hyperammonemia, normal development, osteogenesis imperfecta | X | n.a. | n.a. | ETFDH | c.1084G>A  c.1601C>T | p.G362R  p.P534L | Yotsumoto 2008 [^48^](#_ENREF_48) |
| 110 | f | 22 mo | n.a. | X | - | Vomiting, hypoglycemia, and liver dysfunction, normal development | X | n.a. | n.a. | ETFDH | c.1096C>T  c.1675C>T | p.L366F  p.R559* | Yotsumoto 2008 [^48^](#_ENREF_48) |
| 111 | f | 5 y | n.a. | X | - | Convulsion, hypoglycemia, and liver dysfunction, normal development | X | n.a. | n.a. | ETFDH | c.1096C>T  c.? | p.L366F  p.? | Yotsumoto 2008 [^48^](#_ENREF_48) |
| 112 | f | 13 y | n.a. | X | X | Vomiting, hypotonia, and liver dysfunction | X | n.a. | n.a. | ETFDH | c.524G>A  c.1774T>C | p.R175H  p.C592R | Yotsumoto 2008 [^48^](#_ENREF_48) |
| 113 | m | ca. 40 y | n.a. | - | X | Hypotonia, muscle pain, and CPK elevation | X | n.a. | n.a. | ETFDH | c.1367C>T  c.1367C>T | p.P456L  p.P456L | Yotsumoto 2008 [^48^](#_ENREF_48) |
| 114 | m | 4 mo | n.a. | X | - | No remarkable symptoms but died suddenly at age 3 years | X | n.a. | n.a. | ETFB | c.491G>A  c.IVS5+1G>C | p.R174Q  p.G148-M200del | Yotsumoto 2008 [^48^](#_ENREF_48)/ Colombo 1994 [^49^](#_ENREF_49) |
| 115 | m | 5 mo | n.a. | X | X | Poor feeding, hypoglycemia and liver dysfunction, deceased at age 2 years | X | n.a. | n.a. | ETFB | c.491G>A  c.IVS5+1G>C | p.R174Q  p.G148-M200del | Yotsumoto 2008 [^48^](#_ENREF_48)/ Colombo 1994 [^49^](#_ENREF_49) |
| 116 | m | 1 y | n.a. | X | - | Reye-like illness | X | n.a. | X | ETFA | c.764G>T  c.478delG | p.G255V  p.D160Mfs*4 | Yotsumoto 2008 [^48^](#_ENREF_48)/ Purevjav 2002 [^50^](#_ENREF_50) |
| 117 | m | 43 y | 44 y | - | X | Single episode of myalgia, limb weakness | n.a. | X | n.a. | ETFDH | c.250G>A  c.250G>A | p.A84T  p.A84T | Lan 2010 [^51^](#_ENREF_51) |
| 118 | f | 34 y | 37 y | - | X | Single episode of myalgia, limb weakness, dysphagia | n.a. | X | n.a. | ETFDH | c.250G>A  c.250G>A | p.A84T  p.A84T | Lan 2010 [^51^](#_ENREF_51) |
| 119 | m | 21 y | 26 y | - | X | Single episode of myalgia, limb weakness, dysphagia | n.a. | X | n.a. | ETFDH | c.250G>A  c.250G>A | p.A84T  p.A84T | Lan 2010 [^51^](#_ENREF_51) |
| 120 | f | 20 y | 25 y | X | X | Recurrent episodes of encephalopathy, seizure, hypoglycemia, heart failure,  myalgia, limb weakness, respiratory failure | n.a. | X | n.a. | ETFDH | c.250G>A  c.250G>A | p.A84T  p.A84T | Lan 2010 [^51^](#_ENREF_51) |
| 121 | f | 19 y | 33 y | X | X | Recurrent episodes of vomiting, cardiac arrhythmia, myalgia, limb weakness, dysphagia | n.a. | X | n.a. | ETFDH | c.250G>A  c.250G>A | p.A84T  p.A84T | Lan 2010 [^51^](#_ENREF_51) |
| 122 | f | 7 y | 22 y | X | X | Recurrent episodes of encephalopathy, liver function impairment, lactic acidosis, myalgia, limb weakness, dysphagia, respiratory failure | n.a. | X | n.a. | ETFDH | c.250G>A  c.250G>A | p.A84T  p.A84T | Lan 2010 [^51^](#_ENREF_51) |
| 123 | m | 20 y | 34 y | X | X | Recurrent episodes of vomiting, liver function impairment, myalgia, limb weakness | n.a. | X | n.a. | ETFDH  Additional *PNPLA2-*mutation | c.250G>A  c.524G>A  c.863C>G | p.A84T  p.R175H  p.S288W | Lan 2010 [^51^](#_ENREF_51) |
| 124 | m | 10 y | n.a. | - | X | 6 episodes of severe weakness, dysphagia, weight loss, diarrhea, exercise intolerance in between episodes | X | X | X | ETFDH | c.250G>A  c.1831GNA | p.A84T  p.G611R | Er 2010 [^52^](#_ENREF_52) |
| 125 | f | 8 y | n.a. | X | X | 3 episodes of severe weakness, myalgia, dysphagia, weight loss, abdominal pain; mild weakness in between episodes | X | n.a. | n.a. | ETFDH | c.250G>A  c.1831GNA | p.A84T  p.G611R | Er 2010 [^52^](#_ENREF_52) |
| 126 | f | 11 y | n.a. | - | X | 3 episodes of severe weakness, weight loss; progressive proximal muscle weakness in between episodes | X | X | X | ETFDH | c.250G>A  c.250G>A | p.A84T  p.A84T | Er 2010 [^52^](#_ENREF_52) |
| 127 | m | 12 y | n.a. | - | X | 1 episode of severe weakness, weight loss; exercise intolerance and minimal weakness in after episode | X | X | X | ETFDH | c.250G>A  c.250G>A | p.A84T  p.A84T | Er 2010 [^52^](#_ENREF_52) |
| 128 | m | 21 y | n.a. | - | X | 1 episode of severe weakness, myalgia, dysphagia, weight loss exercise intolerance and minimal weakness after episode | n.a | n.a. | X | ETFDH | c.250G>A  c.250G>A | p.A84T  p.A84T | Er 2010 [^52^](#_ENREF_52) |
| 129 | f | early childhood | 19 y | (X) | X | exercise intolerance since early childhood, 6 episodes of acute pancreatitis, 2 episodes of severe weakness, dysphagia, weight loss, 1 episode of respiratory failure; exercise intolerance and minimal weakness in between episodes | X | X | X | ETFDH | c.250G>A  c.524G>T | p.A84T  p.R175L | Er 2010 [^52^](#_ENREF_52)/ Liang 2009 [^53^](#_ENREF_53)/ Liang 2004 [^54^](#_ENREF_54) |
| 130 | f | 12 y | 35 y | - | X | 2 episodes of severe weakness, weight loss, diarrhea, exercise intolerance in between episodes | X | X | X | ETFDH | c.250G>A  c.524G>T | p.A84T  p.R175L | Er 2010 [^52^](#_ENREF_52)/ Liang 2009 [^53^](#_ENREF_53) |
| 131 | m | 14 y | 20 y | - | X | exercise intolerance at age 14 years, 3 episodes of severe weakness, weight loss, diarrhea; mild weakness in between episodes | -/X | X | X | ETFDH | c.250G>A  c.250G>A | p.A84T  p.A84T | Er 2010 [^52^](#_ENREF_52)/ Liang 2009 [^53^](#_ENREF_53) |
| 132 | f | 10 y | n.a. | X | X | 2 episodes of severe weakness, weight loss, hepatomegaly;  1 episode of coma and cardiopulmonary failure, metabolic acidosis, hyperammonemia and hypoglycemia;  mild weakness in between episodes,  deceased at age 10 years | X | X | ? | ETFDH | c.250G>A  c.380T>A | p.A84T  p.127H | Er 2010 [^52^](#_ENREF_52)/ Liang 2009 [^53^](#_ENREF_53) |
| 133 | m | 8 y | 9.5 y | X | X | 2 episodes of acidotic, nonketotic  hypoglycemic coma, encephalopathy with mutism, ataxia, hypotonia, and bilateral strabismus, slow recovery after episodes, mild cognitive delays,  failure to thrive, mild generalized hypotonia throughout all limbs | - | -/X^◊^ | X | ETFDH | c.79C>T  c.79C>T | p.P27S  p.P27S | Pollard 2010 [^55^](#_ENREF_55) |
| 134 | f | 2.5 y | 2.5 y | - | X | delayed psychomotor development, mild decreased strength in all limbs and trunk and generalized hypotonia, mild truncal ataxia | - | X | n.a. | ETFDH | c.79C>T  c.79C>T | p.P27S  p.P27S | Pollard 2010 [^55^](#_ENREF_55) |
| 135 | m | - | NBS | - | (X) | identified by NBS, mild  speech delays, otherwise asymptomatic at age 2.75 years | -/X | X | n.a. | ETFA | c.937T>A  c.937T>A | p.Y313N  p.Y313N | Pollard 2010 [^55^](#_ENREF_55) |
| 136 | m | 10 mo | NBS | X | - | identified by NBS, but biochemically inconclusive until acute presentation at age 10 months with hypoglycaemia and acidosis | - | -/X | n.a. | n.a. | n.a. | n.a. | Pollard 2010 [^55^](#_ENREF_55) |
| 137 | n.a. | > 8 y, < 22 y | n.a. | n.a. | X | progressive fatigue,  muscular weakness and muscle pain, mild hepatomegaly | n.a. | n.a. | X | n.a. | n.a. | n.a. | Yang 2008 [^56^](#_ENREF_56) |
| 138 | n.a. | > 8 y, < 22 y | n.a. | n.a. | X | progressive fatigue,  muscular weakness and muscle pain, mild hepatomegaly | n.a. | n.a. | X | n.a. | n.a. | n.a. | Yang 2008 [^56^](#_ENREF_56) |
| 139 | n.a. | > 8 y, < 22 y | n.a. | n.a. | X | progressive fatigue,  muscular weakness and muscle pain, mild hepatomegaly | n.a. | n.a. | X | n.a. | n.a. | n.a. | Yang 2008 [^56^](#_ENREF_56) |
| 140 | n.a. | > 8 y, < 22 y | n.a. | n.a. | X | progressive fatigue,  muscular weakness and muscle pain, mild hepatomegaly | n.a. | n.a. | X | n.a. | n.a. | n.a. | Yang 2008 [^56^](#_ENREF_56) |
| 141 | n.a. | > 8 y, < 22 y | n.a. | n.a. | X | progressive fatigue,  muscular weakness and muscle pain, mild hepatomegaly | n.a. | n.a. | X | n.a. | n.a. | n.a. | Yang 2008 [^56^](#_ENREF_56) |
| 142 | n.a. | > 8 y, < 22 y | n.a. | n.a. | X | progressive fatigue,  muscular weakness and muscle pain, mild hepatomegaly | n.a. | n.a. | X | n.a. | n.a. | n.a. | Yang 2008 [^56^](#_ENREF_56) |
| 143 | n.a. | > 8 y, < 22 y | n.a. | n.a. | X | progressive fatigue,  muscular weakness and muscle pain, mild hepatomegaly | n.a. | n.a. | X | n.a. | n.a. | n.a. | Yang 2008 [^56^](#_ENREF_56) |
| 144 | m | 3.5 mo | NBS | X | X | identified by NBS, at age 3.5 months mild hyperammonemia, mild metabolic acidosis and elevated transaminases, at age 4 months hepatomegaly, at age 7 months poor feeding, decreased activity and hypotonia after 2 days of fever, sudden cardiorespiratory arrest during this episode | X | X | n.a. | ETFDH | c.625G>A  c.1852T>C | p.D218N  p.*618Q (original stopcodon lost, results in prolonged protein) | Angle 2008 [^57^](#_ENREF_57) |
| 145 | m | 12 mo | NBS | X | - | At age 12 months acute decompensation associated with Rota-virus gastroenteritis, during this episode acute life-threatening event requiring cardiopulmonary resuscitation, hypoglycaemia, seizures,  full recovery with normal neurological status | X | X | n.a. | ETFDH | c.731T>C  c.814G>A | p.F244S  p.G272R | Angle 2008 [^57^](#_ENREF_57) |
| 146 | m | 2 y | 3 y | X | X | 2 episodes of acute encephalopathy with acidosis and hypoglycemia, Reye's syndrome-like crisis with hepatomegaly; slight reduction in muscle strength and muscle tonus | X | n.a. | X | ETFDH | c.806A>T  c.1448C>T | p.Q269L  p.P483L | Olsen 2007 [^58^](#_ENREF_58)/ Gregersen 1982 [^59^](#_ENREF_59)/ Gregersen 1986 [^60^](#_ENREF_60) |
| 147 | m | 2 y | 13 | X | - | sibling of #146, 1 episode of acute encephalopathy, attack of Reye's syndrome at age 2 years | X | n.a. | n.a. | ETFDH | c.806A>T  c.1448C>T | p.Q269L  p.P483L | Olsen 2007 [^58^](#_ENREF_58)/ Gregersen 1982 [^59^](#_ENREF_59)/ Gregersen 1986 [^60^](#_ENREF_60) |
| 148 | m | 3 y | 7 y | - | X | severe muscle weakness, learning difficulties (IQ 50) | X | X | X | ETFDH | c.244T>C  c.244T>C | p.S82P  p.S82P | Olsen 2007 [^58^](#_ENREF_58)/ Ramos 1995 |
| 149 | f | 14 y | 14 y | X | X | 1 episode of acute encephalopathy with coma and rhabdomyolysis, severe muscle weakness | X | -/X | X | ETFDH | c.1367C>T  c.1367C>T | p.P456L  p.P456L | Olsen 2007 [^58^](#_ENREF_58)/ Henderson 2002 [^61^](#_ENREF_61) |
| 150 | f | n.a. | 19 y | (X) | - | sibling of #149, vomiting | X | -/X | X | ETFDH | c.1367C>T  c.1367C>T | p.P456L  p.P456L | Olsen 2007 [^58^](#_ENREF_58) |
| 151 | f | 9 y | 16 y | X | - | 2 episodes of acute encephalopathy, cyclic vomiting | X | n.a. | X | ETFDH | c.1351G>C  c.1351G>C | p.V451L  p.V451L | Olsen 2007 [^58^](#_ENREF_58) |
| 152 | f | 8 y | 15 y | X | - | identical twin of #151, 2 episodes of acute encephalopathy, cyclic vomiting | X | n.a. | X | ETFDH | c.1351G>C  c.1351G>C | p.V451L  p.V451L | Olsen 2007 [^58^](#_ENREF_58) |
| 153 | f | 13 y | 16 y | - | X | progressive proximal limb and truncal muscle weakness,  lethargy, myalgia, dysphagia, exertional and nocturnal  dyspnoea and choking, generalised muscle wasting | X | X | X | ETFDH | c.334C>T  c.1366C>A | p.H112Y  p.P456T | Olsen 2007 [^58^](#_ENREF_58)/ Beresford 2006 [^62^](#_ENREF_62) |
| 154 | f | 16 y | 17 y | X | X | 2 mild episodes of acute encephalopathy, severe muscle weakness | X | X | X | ETFDH | c.1001T>C  c.1367C>T | p.L334P  p.P456L | Olsen 2007 [^58^](#_ENREF_58) |
| 155 | f | n.a. | 14 y | - | - | sibling of # 154, cyclic vomiting | X | X | X | ETFDH | c.1001T>C  c.1367C>T | p.L334P  p.P456L | Olsen 2007 [^58^](#_ENREF_58) |
| 156 | f | 21 y | 21 y | X | X | 1 episode of acute encephalopathy, severe muscle weakness, cyclic vomiting | X | X | X | ETFDH | c.1445A>T  c.? | p.E482V  p.? | Olsen 2007 [^58^](#_ENREF_58)/ Moore 1998 |
| 157 | f | 22 y | 22 y | - | X | severe muscle weakness | X | X | X | ETFDH | c.51_52insT  c.508G>T | p.A18Cfs*5  p.G170C | Olsen 2007 [^58^](#_ENREF_58) |
| 158 | f | 3 y | 26 y | X | X | 1 episode of acute encephalopathy, severe muscle weakness, cyclic vomiting | X | X | X | ETFDH | c.51_52insT  c.1367C>T | p.A18Cfs*5  p.P456L | Olsen 2007 [^58^](#_ENREF_58) |
| 159 | f | 31 y | 31 y | X | - | 1 episode of acute encephalopathy | X | X | X | ETFDH | c.1060G>T  c.1351G>C | p.354*  p.V451L | Olsen 2007 [^58^](#_ENREF_58)/ Stojanovic 2000 [^63^](#_ENREF_63) |
| 160 | m | 33 y | 42 y | - | X | severe muscle weakness | (X) | n.a. | X | ETFDH | c.?  c.1285G>C | p.A12fs  p.G429R | Olsen 2007 [^58^](#_ENREF_58) |
| 161 | f | 32 y | 33 y | - | X | insulin-dependent diabetes diagnosed at age 14 years, proximal muscle weakness since age 32 years during pregnancy | n.a. | X | X | ETFDH | c.1367C>T  c.1768A>G | p.P456L  p.K590E | Gempel 2007 [^64^](#_ENREF_64)/ Horvath 2006 [^65^](#_ENREF_65) |
| 162 | m | 29 y | 29 y | - | X | premature fatigue, difficulty walking and proximal muscle weakness | n.a. | X | n.a. | ETFDH | c.1130T>C  c.1130T>C | p.L377P  p.L377P | Gempel 2007 [^64^](#_ENREF_64)/ Horvath 2006 [^65^](#_ENREF_65) |
| 163 | m | 13 y | 13 y | - | X | muscle cramps and premature fatigue, no muscle weakness | n.a. | n.a. | n.a. | ETFDH | c.1130T>C  c.1130T>C | p.L377P  p.L377P | Gempel 2007 [^64^](#_ENREF_64) |
| 164 | f | 14 y | 17 y | - | X | sibling of #163, exercise intolerance, premature fatigue and proximal weakness, muscle cramps | n.a. | n.a. | X | ETFDH | c.1130T>C  c.1130T>C | p.L377P  p.L377P | Gempel 2007 [^64^](#_ENREF_64) |
| 165 | f | 12 y | 12 y | - | X | subacute onset of muscle weakness and pain | n.a. | X | X | ETFDH | c.1130T>C  c.1130T>C | p.L377P  p.L377P | Gempel 2007 [^64^](#_ENREF_64) |
| 166 | f | n.a. | n.a. | - | X | younger sibling of #165, similarily affected like sister | n.a. | X | X | ETFDH | c.1130T>C  c.1130T>C | p.L377P  p.L377P | Gempel 2007 [^64^](#_ENREF_64) |
| 167 | f | 12 y | 13 y | - | X | subacute onset of muscle  weakness, myalgia and loss of ambulation within 20 days, 2 siblings had died at ages 6  and 9 months with recurrent vomiting of unknown origin | n.a. | X | X | ETFDH | c.1448C>T  c.1448C>T | p.P483L  p.P483L | Gempel 2007 [^64^](#_ENREF_64) |
| 168 | f | 4 y | 12 y | X | - | recurrent episodes of abdominal discomfort, headache, and biliary vomiting during childhood, once with acidosis, one episode with altered conciousness and hepatomegaly, acidosis, hyperammonemia and ketonuria | X | n.a. | X | n.a. | n.a. | n.a. | Firat 2006 [^66^](#_ENREF_66) |
| 169 | n.a. | 7 mo | 7 mo | X | - | deterioration after a fasting period, severe metabolic acidosis, hyperammonemia, multiorgan failure, deceased at age 7 mo | ∞ | ∞ | n.a. | ETFA | c.72delA  c.797C>T | p.I25*  p.T266M | Schiff 2006 [^67^](#_ENREF_67) |
| 170 | n.a. | - | NBS | - | - | Asymptomatic alive at 14 years of age, normal development,  Brother: fatal Reye  syndrome at 9 months of age | ∞ | ∞ | n.a. | ETFA | c.355_356InsC  c.635T>C | p.L119Pfs*10  p.L212P | Schiff 2006 [^67^](#_ENREF_67) |
| 171 | n.a. | < 1 y | n.a. | - | X | Psychomotor delay, hypotonia, dystonia | ∞ | ∞ | n.a. | ETFA | c.963+1delG  c.? | Exon 11 skipping  p.? | Schiff 2006 [^67^](#_ENREF_67) |
| 172 | n.a. | 2.5 y | 2.5 y | X | - | metabolic coma with hypoglycemia after a 36 hours fast at age 2.5 years, afterwards normal development | ∞ | ∞ | n.a. | ETFA | c.494T>C  c.875_878del | p.V165A  D292Afs*23 | Schiff 2006 [^67^](#_ENREF_67) |
| 173 | n.a. | - | neonatal | - | - | Asymptomatic (screening because of symptomatic sib),  Brother: coma with hypoketotic  hypoglycaemia at age 3 years, good outcome | ∞ | ∞ | n.a. | ETFA | c.797C>T  c.? | p.T266M  p.? | Schiff 2006 [^67^](#_ENREF_67) |
| 174 | n.a. | 3 y | n.a. | X | - | sibling of # 173, coma with hypoketotic hypoglycaemia at age 3 years, good outcome | n.a. | n.a. | n.a. | n.a. | n.a. | n.a. | Schiff 2006 [^67^](#_ENREF_67) |
| 175 | n.a. | 3 y | n.a. | X | - | acute rhabdomyolysis and metabolic acidosis at age 3 years, seizures at age 5 years, normal development | ∞ | ∞ | n.a. | ETFA | c.365G>A  c.365G>A | p.R122K  p.R122K | Schiff 2006 [^67^](#_ENREF_67) |
| 176 | n.a. | 14 y | n.a. | X | - | recurrent vomiting with metabolic acidosis since age 14 years | ∞ | ∞ | n.a. | ETFB | c.571C>T  c.571C>T | p.R191C  p.R191C | Schiff 2006 [^67^](#_ENREF_67) |
| 177 | n.a. | 27 y | n.a. | - | X | muscular exercise intolerance | ∞ | ∞ | X | no mutations of ETFA and ETFB found | n.a. | n.a. | Schiff 2006 [^67^](#_ENREF_67) |
| 178 | m | 49 y | n.a. | - | X | progressive muscle  weakness, after intermittend amelioration of symptoms additional episode of weakness, myalgia, fatigue and dyspnoea at age 54 years, asymptomatic under riboflavin therapy at age 63 years | - | X | X | n.a. | n.a. | n.a. | Gianazza 2006 [^68^](#_ENREF_68) |
| 179 | f | n.a. | neonatal | X | X | diagnosed in neonatal period due to affected older sibling,  apart from mild hypotonia, hepatomegaly and  one episode of hypoglycemia, normal development until age 8 years when decreased muscle  strength and limb-girdle-like muscular hypotrophy were  noted, sudden unexpected death at age 11 years during a long-distance flight,  2 siblings had died during childhood, one with diagnosis of MADD, 2 living siblings #180 and #181 also affected with MADD | X | n.a. | n.a. | n.a. | n.a. | n.a. | Olsen 2005 [^69^](#_ENREF_69) |
| 180 | m | - | neonatal | - | - | sibling of # 179 and 181, diagnosed in neonatal period due to affected older sibling, asymptomatic | X | n.a. | n.a. | ETFDH | c.1074G*>*C  c.1074G*>*C | p.R358S  p.R358S | Olsen 2005 [^69^](#_ENREF_69) |
| 181 | f | n.a. | neonatal | X | - | sibling of # 179 and 180, diagnosed in neonatal period due to affected older sibling, one episode of hypoglycemia and multiorgan failure, full recovery, normal development | X | n.a. | n.a. | ETFDH | c.1074G*>*C  c.1074G*>*C | p.R358S  p.R358S | Olsen 2005 [^69^](#_ENREF_69) |
| 182 | m | n.a. | age at study 31 y | - | X | excercise intolerance, mild muscle weakness, onset after pregnany,  sister with lipid storage myopathy deceased | - | X | n.a. | n.a. | n.a. | n.a. | Russell 2003 [^70^](#_ENREF_70) |
| 183 | m | n.a. | age at study 55 y | - | X | mild excercise intolerance and muscle weakness, insulin-dependent diabetes mellitus | ○ | X | n.a. | n.a. | n.a. | n.a. | Russell 2003 [^70^](#_ENREF_70) |
| 184 | m | n.a. | age at study 15 y | - | X | severe excercise intolerance and muscle weakness, Protein C deficiency | ○ | n.a. | n.a. | n.a. | n.a. | n.a. | Russell 2003 [^70^](#_ENREF_70) |
| 185 | m | n.a. | age at study 25 y | - | X | excercise intolerance and muscle weakness, riboflavin homeostasis defect | ▲ | X | n.a. | n.a. | n.a. | n.a. | Russell 2003 [^70^](#_ENREF_70) |
| 186 | f | n.a. | age at study 35 y | - | X | excercise intolerance and severe muscle weakness, epilepsy | ▲ | X | n.a. | n.a. | n.a. | n.a. | Russell 2003 [^70^](#_ENREF_70) |
| 187 | m | n.a. | age at study 36 y | - | X | severe excercise intolerance, severe tetraplegic myopathy or respiratory insufficiency | - | X | n.a. | n.a. | n.a. | n.a. | Russell 2003 [^70^](#_ENREF_70) |
| 188 | f | n.a. | age at study 41 y | - | X | severe excercise intolerance, severe tetraplegic myopathy or respiratory insufficiency | ▲ | n.a. | n.a. | n.a. | n.a. | n.a. | Russell 2003 [^70^](#_ENREF_70) |
| 189 | m | 4 mo | 4 mo | X | X | at age 4 months hypotonia,  hepatomegaly, stridor, swallowing dysfunction, hyperammonaemia, and high transaminases and creatine kinase enzyme activity, until age 2 years nearly age-appropriate development, at 2 years difficulty walking with spasticity of his right leg  and arm, persistent clonus, progressive to complete spastic quadriplegia, leukodystrophy in brain MRI, good response to therapy with sodium-D,L-3-hydroxybutyrate | X■ | X■ | n.a. | ETFDH | c.?  c.? | p.G381R  p.G381R | Van Hove 2003 [^71^](#_ENREF_71) |
| 190 | f | 5 mo | 5 mo | - | X | heart failure at age 5 months, severe dilated cardiomyopathy with left-ventricular hypertrophy, fractional shortening of 6%, severe muscular hypotonia/ weakness, good response to therapy with sodium-D,L-3-hydroxybutyrate,  three previous siblings had  died in infancy of MADD | X | X | n.a. | n.a. | n.a. | n.a. | Van Hove 2003 [^71^](#_ENREF_71) |
| 191 | f | 18 mo | 18 mo | X | - | episode of hypotonia and decreased level of  consciousness 12 h after the beginning of a mild gastroenteritis, hypoglycemia and mild hyperammonemia, normal development at age 3 years | X | - | n.a. | ETFB  ETFDH | c.124T>C  c.604_606AAGdel  c.1989A>C (polymorphic in the Danish population) | p.C42R  p.K202del | Van Hove 2003 [^71^](#_ENREF_71) |
| 192 | m | 10 mo | n.a. | X | X | failure to thrive, hypotonia, and gross motor delays, with episodes of ketotic hypoglycemia associated with infections | X | n.a. | n.a. | n.a. | n.a. | n.a. | Loehr 1990 [^72^](#_ENREF_72) |
| 193 | m | n.a. | 2 mo | n.a. | n.a. | died at home during first year of life | X | n.a. | n.a. | n.a. | n.a. | n.a. | Loehr 1990 [^72^](#_ENREF_72) |
| 194 | f | childhood | 19 y | X | X | progressive weakness and intolerance to fasting, at age 18 years marked fatigue, muscle weakness, and hepatomegaly, which cleared spontaneously, at age 19 years episode of marked muscle weakness and hypoglycaemia, proximal upper and lower limb myopathy, hepatomegaly, and mild hyperammonemia, lipid storage myopathy, death from adult respiratory distress syndrome  secondary to aspiration | X | n.a. | X | n.a. | n.a. | n.a. | Loehr 1990 [^72^](#_ENREF_72) |
| 195 | f | 5 mo | 9 mo | X | - | episodes of vomiting, lethargy, and pallor at 5 and 6 months of age, second episode with respiratory arrest, recovered | -/X | n.a. | n.a. | n.a. | n.a. | n.a. | Loehr 1990 [^72^](#_ENREF_72) |
| 196 | f | 23 y | 23 y | - | X | progressive weakness during pregnancy and preterm labor, slurred speech, muscle cramps and myalgias, decreased appetite, weight loss, increased weakness after delivery | X | -/X | X | no ETFDH mutation detectable | n.a. | n.a. | Mareska 2003 [^24^](#_ENREF_24) |
| 197 | f | - | NBS | - | - | asymptomatic, normal development at age 3.9 years, 3 siblings had died, one of them with proven MADD | - | X | n.a. | n.a. | n.a. | n.a. | Abdenur 2001 [^73^](#_ENREF_73) |
| 198 | m | 15 y | 16 y | - | X | myalgia and weakness after moderate physical excercise | n.a. | n.a. | X | n.a. | n.a. | n.a. | Monici 1998 [^74^](#_ENREF_74) |
| 199 | m | 23 y | 47 y | X | X | progressive muscular fatigability with episodes of nausea and vomiting, periodic worsening of myalgia and weakness | n.a. | n.a. | X | n.a. | n.a. | n.a. | Monici 1998 [^74^](#_ENREF_74) |
| 200 | m | 8 mo | 8 mo | X | - | presentation with fits and hepatomegaly, rapid recovery, normal development at age 8 years | X | n.a. | - | n.a. | n.a. | n.a. | Burns 1998 [^75^](#_ENREF_75) |
| 201 | f | n.a. | n.a. | n.a. | n.a. | “mild form of the disorder“ | X | n.a. | n.a. | n.a. | n.a. | n.a. | Burns 1998 [^75^](#_ENREF_75) |
| 202 | f | 2 mo | 2 mo | n.a. | X | epilepsy | X | n.a. | n.a. | n.a. | n.a. | n.a. | Pang 1997 [^76^](#_ENREF_76) |
| 203 | m | < 2 y | 2.3 y | X | X | high frequency of respiratory infections and diarrhoea during first 2 years of life, progressive generalized hypotonia with hepatomegaly, at age 2.3 years coma with generalized hypotonia, hepatomegaly, hypoglycaemia, lactatemia and mild hyperammonemia, lipid storage myopathy, sister had died at age 1 year | X | n.a. | n.a. | n.a. | n.a. | n.a. | Jakobs 1997 [^77^](#_ENREF_77)/ Poll-The 1988 [^78^](#_ENREF_78) |
| 204 | f | 22 mo | 5 y | X | X | at age 22 months stroke with mild cerebral vascular accident, right hemiplegia, pyramidal signs, moderate static and kinetic ataxia, psychomotor delay, epilepsy; metabolic decompensation with hypoglycaemia, acidosis, drowsiness and ataxia at age 5 years | -/X | n.a. | X | n.a. | n.a. | n.a. | Fontaine 1996 [^79^](#_ENREF_79)/ Vallée 1994 [^80^](#_ENREF_80) |
| 205 | m | < 6 y | < 6 y | n.a. | n.a. | „mild type of MADD“ | X | n.a. | n.a. | n.a. | n.a. | n.a. | Poorthuis 1993 [^81^](#_ENREF_81) |
| 206 | m | 9 mo | 9 mo | X | - | hypoglycaemic coma with acidosis, hepatomegaly, hepatic steatosis, died from a Reye's syndrome at age 9 months, fatty vacuolisation of myocardiac fibres at autopsy | X | n.a. | n.a. | n.a. | n.a. | n.a. | Guffon 1993 [^82^](#_ENREF_82) |
| 207 | m | - | neonatal | - | - | sibling of #206, asymptomatic at age 6 months | X | n.a. | n.a. | n.a. | n.a. | n.a. | Guffon 1993 [^82^](#_ENREF_82) |
| 208 | f | 18 y | n.a. | X | X | muscle pain, intolerance to exercise and episodes of nausea and vomiting, blood glucose occasionally low | (X) | n.a. | X | n.a. | n.a. | n.a. | Peluchetti 1991 [^35^](#_ENREF_35) |
| 209 | m | 13 y | 13 y | - | X | easy fatigability and exercise intolerance, severe generalized muscle weakness and wasting | (X) | n.a. | X | n.a. | n.a. | n.a. | Peluchetti 1991 [^35^](#_ENREF_35) |
| 210 | m | 4 y | n.a. | - | X | easy fatigability and exercise intolerance, muscle pain, mild generalized muscle weakness and wasting | (X) | n.a. | X | n.a. | n.a. | n.a. | Peluchetti 1991 [^35^](#_ENREF_35) |
| 211 | f | 25 y | 25 y | - | X | progressive proximal muscle weakness, pretibial edema, progressive fatigue,  with thigh and calf weakness, exercise intolerance | X | n.a. | X | n.a. | n.a. | n.a. | Gilkeson 1988 [^83^](#_ENREF_83)/ Mareska 2003 [^24^](#_ENREF_24) |
| 212 | m | n.a. | 7 y | X | - | recurrent attacks of metabolic decompensation with acidosis and hypoglycemia, at times proceeding to coma and requiring intensive care support, 2-3 metabolic crises per year, especially after meals rich in fat or protein, hepatomegaly | X | X | X | n.a. | n.a. | n.a. | Al-Essa 2000 [^84^](#_ENREF_84) |
| 213 | f | n.a. | 1 y | X | - | recurrent attacks of metabolic decompensation with acidosis and hypoglycemia, at times proceeding to coma and requiring intensive care support, 2-3 metabolic crises per year, especially after meals rich in fat or protein, hypochromic, microcytic anemia | X | X | X | n.a. | n.a. | n.a. | Al-Essa 2000 [^84^](#_ENREF_84) |
| 214 | f | childhood, n.a. | 13 y | - | X | slow runner, easy  fatigability since her preschool years, at age 12 years intermittent nausea, vomiting, and anorexia, at age 13 years general fatigue, muscle weakness, exercise intolerance, weight loss and depression, Brain MRI: disseminated high-intensity areas in the periventricular white matter and in the splenium of the corpus callosum | X | -? | X | ETFDH | c.524G>A  c.1774T>G | p.R175H  p.C592R | Ishii 2010 [^85^](#_ENREF_85) |
| 215 | f | 24 y | n.a. | - | X | muscle weakness, exercise intolerance | n.a. | X | X | ETFDH | c.1227A>C  c.872T>G | p.L409F  p.V291G | Wen 2010 [^86^](#_ENREF_86) |
| 216 | f | 23 y | n.a. | X | X | episodes of vomiting, muscle weakness, exercise intolerance | n.a. | X | X | ETFDH | c.1227A>C  c.872T>G | p.L409F  p.V291G | Wen 2010 [^86^](#_ENREF_86) |
| 217 | m | 13 y | n.a. | X | X | episodes of vomiting, muscle weakness, exercise intolerance | n.a. | X | X | ETFDH | c.1227A>C  c.393G>C | p.L409F  p.W131C | Wen 2010 [^86^](#_ENREF_86) |
| 218 | m | 12 y | n.a. | X | X | episodes of vomiting, muscle weakness, exercise intolerance | X | X | X | ETFDH | c.1227A>C  c.1399G>C | p.L409F  p.G467R | Wen 2010 [^86^](#_ENREF_86) |
| 219 | f | 6 y | n.a. | - | X | muscle weakness, exercise intolerance | n.a. | X | X | ETFDH | c.242T>C  IVS4-9T>C | p.L81P  truncated | Wen 2010 [^86^](#_ENREF_86) |
| 220 | m | 15 y | n.a. | - | X | muscle weakness, exercise intolerance | X | X | X | n.a. | no mutations of ETFA, ETFB and ETFDH found |  | Wen 2010 [^86^](#_ENREF_86) |
| 221 | f | 48 y | n.a. | X | X | episodes of vomiting, muscle weakness, exercise intolerance | n.a. | X | X | ETFDH | c.770A>G  c.? | p.Y257C  p.? | Wen 2010 [^86^](#_ENREF_86) |
| 222 | m | 19 y | n.a. | - | X | muscle weakness, exercise intolerance | n.a. | X | X | ETFDH | c.1227A>C  IVS12-3C>G | p.L409F  p.? | Wen 2010 [^86^](#_ENREF_86) |
| 223 | f | 17 y | n.a. | - | X | muscle weakness, exercise intolerance | X | X | X | ETFDH | c.380T>G  c.1531G>A | p.L127R  p.D511N | Wen 2010 [^86^](#_ENREF_86) |
| 224 | f | 22 y | n.a. | - | X | muscle weakness, exercise intolerance | X | X | X | ETFDH | c.770A>G  c.872T>G | p.Y257C  p.V291G | Wen 2010 [^86^](#_ENREF_86) |
| 225 | f | 33 y | n.a. | - | X | muscle weakness, exercise intolerance | X | X | X | ETFDH | c.770A>G  c.973del312 | p.Y257C  p.325del48 | Wen 2010 [^86^](#_ENREF_86) |
| 226 | f | 17 y | n.a. | X | X | episodes of vomiting, muscle weakness, exercise intolerance | X | X | X | ETFDH | c.770A>G  c.1084G>A  c.1399G>A | p.Y257C  p.G362R  p.G467R | Wen 2010 [^86^](#_ENREF_86) |
| 227 | f | 16 y | n.a. | X | X | episodes of vomiting, muscle weakness, exercise intolerance | X | X | X | ETFDH | c.770A>G  c.770A>G | p.Y257C  p.Y257C | Wen 2010 [^86^](#_ENREF_86) |
| 228 | f | 15 y | n.a. | - | X | muscle weakness, exercise intolerance | X | X | X | ETFDH | c.1227A>C  c.389A>T | p.L409F  p.D130V | Wen 2010 [^86^](#_ENREF_86) |
| 229 | f | 63 y | n.a. | - | X | muscle weakness, exercise intolerance | X | X | X | ETFDH | IVS3+1G>A  - | p.?  - | Wen 2010 [^86^](#_ENREF_86) |
| 230 | f | 23 y | n.a. | X | X | episodes of vomiting, muscle weakness, exercise intolerance | X | X | X | ETFDH | c.1212T>C  c.? | p.M404T  p.? | Wen 2010 [^86^](#_ENREF_86) |
| 231 | f | 10 y | n.a. | - | X | muscle weakness, exercise intolerance | X | X | X | ETFDH | c.1436G>C  c.1395T>G | p.R479T  p.Y465* | Wen 2010 [^86^](#_ENREF_86) |
| 232 | m | 17 y | n.a. | X | X | episodes of vomiting, muscle weakness, exercise intolerance | - | - | X | ETFDH | c.715G>A  c.1395T>G | p.A239T  p.Y465* | Wen 2010 [^86^](#_ENREF_86) |
| 233 | f | 22 y | n.a. | - | X | muscle weakness, exercise intolerance | - | - | X | ETFDH | c.1227A>C  c.? | p.L409F  p.? | Wen 2010 [^86^](#_ENREF_86) |
| 234 | f | 16 y | 16 y | X | - | at age 16 years altered mental status, acidosis, hyperammonemia and ketosis during urinary tract infection, narcolepsy and cataplexy since age 13 years | X | X | X | n.a. | n.a. | n.a. | Williams 2008 [^87^](#_ENREF_87) |
| 235 | f | childhood | 19 y | X | X | episodes of mild weakness, easy fatigability and bouts of nausea during childhood, since age 19 years recurrent episodes of hypoglycemia, hypoglycemic coma, fatty infiltration of the liver, hepatomegaly, hepatic dysfunction, weakness and wasting of the proximal muscles | X | - | n.a. | n.a. | n.a. | n.a. | Dusheiko 1979 [^88^](#_ENREF_88)/ Mareska 2003 [^24^](#_ENREF_24) |
| 236 | f | 14 y | 17 y | X | X | episode of unexplained vomiting and abdominal pains at age 14 years, muscular weakness, progressive lipid storage myopathy | X | n.a. | X | n.a. | n.a. | n.a. | de Visser 1986 [^89^](#_ENREF_89) |
| 237 | n.a. | < 1 y, > 1 mo | n.a. | - | X | skeletal muscle involvement, deceased in childhood | n.a. | n.a. | n.a. | n.a. | n.a. | n.a. | Tamaoki 2002 [^90^](#_ENREF_90) |
| 238 | n.a. | < 1 y, > 1 mo | n.a. | - | X | skeletal muscle involvement, mildly handicapped | n.a. | n.a. | n.a. | n.a. | n.a. | n.a. | Tamaoki 2002 [^90^](#_ENREF_90) |
| 239 | n.a. | < 1 y, > 1 mo | n.a. | - | X | skeletal muscle involvement, normal or subnormal development | n.a. | n.a. | n.a. | n.a. | n.a. | n.a. | Tamaoki 2002 [^90^](#_ENREF_90) |
| 240 | n.a. | < 1 y, > 1 mo | n.a. | X | - | encephalopathy, Reye-like presentation or SIDS, deceased | n.a. | n.a. | n.a. | n.a. | n.a. | n.a. | Tamaoki 2002 [^90^](#_ENREF_90) |
| 241 | n.a. | 1 y | n.a. | X | - | encephalopathy, Reye-like presentation or SIDS, normal or subnormal development | n.a. | n.a. | n.a. | n.a. | n.a. | n.a. | Tamaoki 2002 [^90^](#_ENREF_90) |
| 242 | n.a. | - | 1.5 y | - | - | asymptomatic, normal development | n.a. | n.a. | n.a. | n.a. | n.a. | n.a. | Tamaoki 2002 [^90^](#_ENREF_90) |
| 243 | n.a. | 5 y | n.a. | X | n.a. | encephalopathy, Reye-like presentation or SIDS, severely handicapped | n.a. | n.a. | n.a. | n.a. | n.a. | n.a. | Tamaoki 2002 [^90^](#_ENREF_90) |
| 244 | n.a. | 15 y | n.a. | - | X | skeletal muscle involvement, normal or subnormal development | n.a. | n.a. | n.a. | n.a. | n.a. | n.a. | Tamaoki 2002 [^90^](#_ENREF_90) |
| 245 | f | n.a. | 5 mo | - | X | muscle weakness, hepatomegaly | X | n.a. | n.a. | ETFDH | c.1519T>G | p.Y507D | Ohkuma 2009 [^91^](#_ENREF_91) |
| 246 | m | n.a. | 6 mo | - | X | muscle weakness, hepatomegaly, normal development after  treatment | X | n.a. | X | ETFDH | c.1208C>T  c.1208C>T | p.A403V  p.A403V | Ohkuma 2009 [^91^](#_ENREF_91) |
| 247 | m | n.a. | 11 mo | X | X | vomiting, hypertrophic  cardiomyopathy, died at 2 years of age due to pulmonary alveolar bleeding | X | n.a. | n.a. | ETFA | c.284T>G  c.284T>G | p.L95W  p.L95W | Ohkuma 2009 [^91^](#_ENREF_91) |
| 248 | f | n.a. | 13.3 y | - | X | progressive muscle weakness | X | n.a. | X | ETFDH | c.524G>A  c.1774T>G | p.R175H  p.C592R | Ohkuma 2009 [^91^](#_ENREF_91) |
| 249 | n.a. | n.a. | n.a. | X | - | “near-miss”- SIDS at age 2 months, lethargy, acrid breath | ○ | n.a. | X | n.a. | n.a. | n.a. | Harpey 1987 [^92^](#_ENREF_92) |
| 250 | f | 18 mo | 18 mo | X | - | episode of hypotonia and decreased level of  consciousness after mild gastroenteritis, hypoglycaemia, normal development | X | n.a. | X | ETFB | c.124T> C  c.604_606AAGdel | p.C42R  p.K202del | Curcoy 2003 [^93^](#_ENREF_93) |
| 251 | f | 18 y | 21 y | - | X | fatigability, muscular weakness, lipid storage myopathy | X | X | X | ETFDH | c.250G>A  c.524G>A | p.A84T  p.R175H | Maillart 2010 [^94^](#_ENREF_94) |
| 252 | m | 18 y | 43 y | X | X | at age 18 years recurrent vomiting and impaired vigilance, muscular weakness, at age 28 years exercise intolerance, shortness of breath, muscle pain, muscular weakness and weight loss of 13 kg, amelioration under carnitine treatment, at age 43 years third episode of muscular symptoms | n.a. | X | X | ETFDH | c.877C>G  c.1691-3C>G | p.H293D  p.? | Maillart 2010 [^94^](#_ENREF_94) |
| 253 | m | 9 mo | n.a. | X | - | episodes of hypoglycemia, vomiting | X° | n.a. | X | n.a. | n.a. | n.a. | Green 1985 [^95^](#_ENREF_95)/ Green 1991 [^96^](#_ENREF_96)/ Manning 1990 [^28^](#_ENREF_28) |
| 254 | m | 7 mo | 7 mo | X | - | peculiar sweat sock breath odour, altered sensorium, hypotonia, hypoglycemia, elevated anion gap | - | n.a. | n.a. | n.a. | n.a. | n.a. | Mumtaz 2010 [^15^](#_ENREF_15) |
| 255-325 | 47 m  24 f | average 25.0 y (4-36 y) | n.a. | 19/90 | 90/90 | 87/90 (96.7%) muscle weakness,  3/90 (3.4%) respiratory failure,  2/90 (2.2%) exercise intolerance,  19/90 (21.1%) vomiting/diarrhea,  9/90 (10.0%) fatty liver,  2/90 (2.2%) palpitation or short of breath,  1/90 (1.1) cardiac diastolic dysfunction | 34/41  (82.9%),  normal in 7/41 (17.1%) | 42/48 (87.5%) | 90/90 (100%) | ETFDH | 1 patient:  c.1227A>C  c.872T>G  1 patient:  c.770A>G  c.1448C>T  1 patient:  c.1227A>C  c.503A>G  1 patient:  c.1227A>C  c.692T>C  1 patient:  c.770A>G  c.1395T>G  1 patient:  c.1227A>C  c.524G>T  1 patient:  c.770A>G  c.1099A>G  1 patient:  c.389A>T  c.361_362 insT  1 patient:  c.1227A>C  c.389A>T  1 patient:  c.236C>G  c.1281_1282del  1 patient:  c.389A>T  c.835T>C  1 patient:  c.353G>T  c.1657T>C  1 patient:  c.1227A>C  c.213_215del  1 patient:  c.770A>G  c.1281_1282del  1 patient:  c.389A>T  c.835T>C  1 patient:  c.770A>G  c. -75A>G  1 patient:  c.770A>G  c.1531G>A  c.250G>A  1 patient:  c.389A>T  c.1395T>G  1 patient:  c.1227A>C  c.250G>A  1 patient:  c.770A>G  c.872T>G  1 patient:  c.770A>G  c.1372_1375del  1 patient:  c.250G>A  c.524G>A  1 patient:  c.256C>T  c.175+2T>C  1 patient:  c.770A>G  c.1099A>G  1 patient:  c.250G>A  c.528G>C  1 patient:  c.770A>G  c.1027T>C  1 patient:  c.770A>G  c.1026G>T  1 patient:  c.242T>C  c. 606+7A>G  1 patient:  c.389A>T  c.1395T>G  1 patient:  c.389A>T  c.503A>G  1 patient:  c.389A>T  c.1205C>T  1 patient:  c.770A>G  c.1448C>T  1 patient:  c.250G>A  c.389A>T  6 patients:  c.250G>A  c.250G>A  1 patient:  c.1227A>C  c.1227A>C  1 patient:  c.191G>A  c.191G>A  1 patient:  c.1454C>G  c.1454C>G  1 patient:  c.770A>G  c.1378G>T  1 patient:  c.409C>T  c.1448C>T  1 patient:  c.433G>C  c.949C>A  1 patient:  c.770A>G  c.1763A>G  1 patient:  c.1212T>C  c.1227A>C  1 patient:  c.389A>T  c.872T>G  1 patient:  c.405+1G>T  c.770A>G  1 patient:  c.250G>A  c.998A>G  1 patient:  c.524G>T  c.770A>G  1 patient:  c.226G>A; c.227C>A  c.250G>A  1 patient:  c.389A>T  c.1084G>A  1 patient:  c.770A>G  c.1395T>G  1 patient:  c.1281_1282del  c.1227A>C  1 patient:  c.172G>A  c.1454C>G  1 patient:  c.1395T>G  c.1744A>T  1 patient:  c.250G>A  c.1227A>C  1 patient:  c.250G>A  c.976G>C  1 patient:  c.3G>C  c.770A>G  1 patient:  c.349G>C  c.1227A>C  1 patient:  c.770A>G  c.1691-3C>G  1 patient:  c.1454C>G  c.1773_1774del  1 patient:  c.1212T>C  c.1227A>C  1 patient:  c.152G>A  c.250G>A  6 patients with only 1of the following mutations identified:  c.770A>G  (2 patients)  c.1227A>C  (1 patient)  c.251C>T  (1 patient)  c.389A>T  (1 patient)  c.1395T>G  (1 patient) | p.L409F  p.V291G  p.Y257C  p.P483L  p.L409F  p.N168S  p.L409F  p.F231S  p.Y257C  p.Y465*  p.L409F  p.R175L  p.Y257C  p.N367D  p.D130V  p.P121Lfs*5  p.L409F  p.D130V  p.A79G  p.I428Rfs*6  p.D130V  p.W279R  p.C118F  p.Y553H  p.L409F  p.V72del  p.Y257C  p.I428Rfs*6  p.D130V  p.W279R  p.Y257C  Transcript↓  p.Y257C  p.D511N  p.A84T  p.D130V  p.Y465*  p.L409F  p.A84T  p.Y257C  p.V291G  p.Y257C  p. C458Tfs*10  p.A84T  p.R175H  p.R86C  Truncated  p.Y257C  p.N367D  p.A84T  p.L176F  p.Y257C  p.W343R  p.Y257C  p.R342S  p.L81P  Truncated  p.D130V  p.Y465*  p.D130V  p.N168S  p.D130V  p.T402I  p.Y257C  p.P483L  p.A84T  p.D130V  p.A84T  p.A84T  p.L409F  p.L409F  p.R64K  p.R64K  p.T485S  p.T485S  p.Y257C  p.G460*  p.P137S  p.P483L  p.D145H  p.P317T  p.Y257C  p.H588R  p.M404T  p.L409F  p.D130V  p.V291G  Truncated  p.Y257C  p.A84T  p.Y333C  p.R175L  p.Y257C  p.A76K  p.A84T  p.D130V  p.G362R  p.Y257C  p.Y465*  p.I428Rfs*6  p.L409F  p.E58K  p.T485S  p.Y465*  p.N582Y  p.A84T  p.L409F  p.A84T  p.G326R  p.M1?  p.Y257C  p.A117P  p.L409F  p.Y257C  truncated  p.T485S  p.C592*  p.M404T  p.L409F  p.R51Q  p.A84T  p.Y257C  p.L409F  p.A84V  p.D130V  p.Y465* | Xi 2013 [^97^](#_ENREF_97) |
| 326 | m | 22 y | 22 y | X | X | muscular weakness, chronic diarrhea, episodic nausea and vomiting, elevated transaminases, elevated CK and LDH, hepatic steatosis with hepatomegaly, acute decompensation with hypoglycaemia, somnolence and pancreatitis, lipid storage myopathy | n.a. | n.a. | X | ETFDH | c.1130T>C  c.1130T>C | p.L377P  p.L377P | Scheicht 2013 [^98^](#_ENREF_98) |
| 327 | n.a. | 17 y | 17 y | X | X | weakness, dyspnea and rhabdomyolysis | n.a. | X | X | ETFDH | c.251C>T  c.251C>T | p.A84V  p.A84V | Chien 2014 [^6^](#_ENREF_6) |
| 328 | f | - | 2.9 y | - | - | diagnosed by family screening, asymptomatic | n.a. | X | n.a. | ETFDH | c.295C>G  ? | p.R99G  ? | Chien 2014 [^99^](#_ENREF_99) |
| 329 | f | 25 y | 25 y | X | X | fluctuating weakness, CK 20.000 U/l, elevated liver enzymes, rhabdomyolysis, progressive quadriparesis with involvement of respiratory muscles, lipid storage myopathy, several crises with acute quadriparesis,  one-week period of severe vomiting with a 10 kg weight loss at age 24, 2 uneventful pregnancies, no weakness during religiously motivated fasting periods | X^#&^ | (X^&^) | X | ETFDH | c.1544G>T  c.1544G>T | p.S515I  p.S515I | Rosenbohm 2014 [^100^](#_ENREF_100) |
| 330-350 | 13 m  8 f | n.a. | n.a. | - | X | proximal muscle weakness, exercise intolerance, elevated CK levels, no episodic encephalopathy | X 9/21,  n.a. 12/21 | X 5/21,  n.a.  15/21,  low C0 in 1/21 | X (21/21) | ETFDH | c.1295 T > A  c. 1528C > T  c.770A > G  c. −75A > G  c.770A > G  c.1531G > A  c.250G > A  c.389A > T  c.1395 T > G  c.1227A > C  c.250G > A  c.770A > G  c.872 T > G  c.770A > G  c.1372_1375del  c.389A > T  r.0?  c.250G > A  c.524G > A  c. 256C > T  c.175 + 2 T > C  c.250G > A  c.528G > C  c.770A > G  c.T1027C  c.242 T > C  c.606 + 7A > G  c.389A > T  c.503A > G  c.389A > T  c.360_361 insT  c.1227A > C  c.389A > T  c.236C > G  c.1281_1282del  c.389A > T  c.835 T > C  c.251C > T  r.0?  c.1227A > C  c.210_212del  c.1227A > C  r.0? | p.V432G  p.P510S  p.Y257C  Transcript↓  p.Y257C  p.D511N  p.A84T  p.D130V  p.Y465*  p.L409F  p.A84T  p.Y257C  p.V291G  p.Y257C  p. 467*  p.D130V  p.0  p.A84T  p.R175H  p.R86C  truncated  p.A84T  p.L176F  p.Y257C  p.W343R  p.L81P  truncated  p.D130V  p.N168S  p.D130V  p.125*  p.L409F  p.D130V  p.A79G  p.433*  p.D130V  p.W279R  p.A84V  p.0  p.L409F  p.C71del  p.L409F  p.0 | Wen 2013 [^101^](#_ENREF_101) |

f, female; m, male; d, day(s); mo, month(s); y, year(s), n.a., not available, ?, unknown

*OA under riboflavine therapy normal in 12 of 12 cases

^§^ Plasma acylcarnitines were variable, being both normal and in one instance suggestive of very-long chain acyl-CoA dehydrogenase deficiency

^$^ Abnormal carnitine-ester profiles were seen in plasma samples after a prolonged exercise test

^#^ Metabolites indicative for MADD during metabolic decompensation

^&^ normal(ized) under treatment with riboflavin

^◊^  AC profile post-illness normal, however, a significant plasma carnitine deficiency was noted. After carnitine supplementation an acyl-

carnitine profile consistent with multiple acyl-CoA dehydrogenase deficiency (moderate elevations in plasma short, medium, and long chain acylcarnitines) was observed.

∞ enzyme defect was initially indicated by a characteristic acylcarnitine profile in plasma and/or by a characteristic profile of organic acids in urine using tandem mass spectrometry (MS/MS) or gas chromatography–mass spectrometry (GC–MS), respectively

○ only increased excretion of ethylmalonic acid (and adipic acid) reported

▲ only increased excretion of glutaric acid reported

■ reported as “characteristic metabolites of MADD“

° extremely variable pattern of organic acids

1. Fitzgerald M, Crushell E, Hickey C. Cyclic vomiting syndrome masking a fatal metabolic disease. Eur J Pediatr. 2013 May;172(5):707-10.

2. Trakadis Y, Kadlubowska D, Barnes R, et al. Pregnancy of a patient with multiple Acyl-CoA dehydrogenation deficiency (MADD). Mol Genet Metab. 2012 Aug;106(4):491-4.

3. Zhao ZN, Bao MX, Ma GT, et al. A case of late-onset riboflavin responsive multiple acyl-CoA dehydrogenase deficiency with novel mutations in ETFDH gene. CNS Neurosci Ther. 2012 Nov;18(11):952-4.

4. Izumi R, Suzuki N, Nagata M, et al. A case of late onset riboflavin-responsive multiple acyl-CoA dehydrogenase deficiency manifesting as recurrent rhabdomyolysis and acute renal failure. Intern Med. 2011;50(21):2663-8.

5. Er TK, Chen CC, Liu YY, et al. Computational analysis of a novel mutation in ETFDH gene highlights its long-range effects on the FAD-binding motif. BMC Struct Biol. 2011;11:43.

6. Chien YH, Lee NC, Chao MC, et al. Fatty Acid oxidation disorders in a chinese population in taiwan. JIMD Rep. 2013;11:165-72.

7. Sugai F, Baba K, Toyooka K, et al. Adult-onset multiple acyl CoA dehydrogenation deficiency associated with an abnormal isoenzyme pattern of serum lactate dehydrogenase. Neuromuscul Disord. 2012 Feb;22(2):159-61.

8. Rosa M, Pascarella A, Parenti G, et al. Developmental evolution in a patient with multiple acyl-coenzymeA dehydrogenase deficiency under pharmacological treatment. Eur J Paediatr Neurol. 2012 Mar;16(2):203-5.

9. Lammer AB, Rolinski B, Ahting U, Heuss D. Multiple acyl-CoA-dehydrogenase deficiency (MADD)--a novel mutation of electron-transferring-flavoprotein dehydrogenase ETFDH. J Neurol Sci. 2011 Aug 15;307(1-2):166-7.

10. Wang ZQ, Chen XJ, Murong SX, Wang N, Wu ZY. Molecular analysis of 51 unrelated pedigrees with late-onset multiple acyl-CoA dehydrogenation deficiency (MADD) in southern China confirmed the most common ETFDH mutation and high carrier frequency of c.250G>A. J Mol Med (Berl). 2011 Jun;89(6):569-76.

11. Kaminsky P, Acquaviva-Bourdain C, Jonas J, et al. Subacute myopathy in a mature patient due to multiple acyl-coenzyme A dehydrogenase deficiency. Muscle Nerve. 2011 Mar;43(3):444-6.

12. Wolfe LA, He M, Vockley J, et al. Novel ETF dehydrogenase mutations in a patient with mild glutaric aciduria type II and complex II-III deficiency in liver and muscle. J Inherit Metab Dis. 2010 Nov 19.

13. Wasant P, Kuptanon C, Vattanavicharn N, et al. Glutaric aciduria type 2, late onset type in Thai siblings with myopathy. Pediatr Neurol. 2010 Oct;43(4):279-82.

14. Lee HC, Lai CK, Siu TS, et al. Role of postmortem genetic testing demonstrated in a case of glutaric aciduria type II. Diagn Mol Pathol. 2010 Sep;19(3):184-6.

15. Mumtaz HA, Gupta V, Singh P, Marwaha RK, Khandelwal N. MR imaging findings of glutaric aciduria type II. Singapore Med J. 2010 Apr;51(4):e69-71.

16. Koppel S, Gottschalk J, Hoffmann GF, Waterham HR, Blobel H, Kolker S. Late-onset multiple acyl-CoA dehydrogenase deficiency: a frequently missed diagnosis? Neurology. 2006 Oct 24;67(8):1519.

17. Takken T, Custers J, Visser G, Dorland L, Helders P, de Koning T. Prolonged exercise testing in two children with a mild Multiple Acyl-CoA-Dehydrogenase deficiency. Nutr Metab (Lond). 2005 May 20;2(1):12.

18. Olsen RK, Pourfarzam M, Morris AA, et al. Lipid-storage myopathy and respiratory insufficiency due to ETFQO mutations in a patient with late-onset multiple acyl-CoA dehydrogenation deficiency. J Inherit Metab Dis. 2004;27(5):671-8.

19. Turnbull DM, Bartlett K, Eyre JA, et al. Lipid storage myopathy due to glutaric aciduria type II: treatment of a potentially fatal myopathy. Dev Med Child Neurol. 1988 Oct;30(5):667-72.

20. Liang Y, Liu L, Wei H, Luo XP, Wang MT. [Late-onset riboflavin-responsive multiple acyl-CoA dehydrogenase deficiency (glutaric aciduria type II)]. Zhonghua Er Ke Za Zhi. 2003 Dec;41(12):916-20.

21. Grice AS, Peck TE. Multiple acyl-CoA dehydrogenase deficiency: a rare cause of acidosis with an increased anion gap. Br J Anaesth. 2001 Mar;86(3):437-41.

22. Tojo M, Gunji T, Yamaguchi S, Shimizu N, Koga Y, Nonaka I. [A case of riboflavin-responsive multiple acyl-CoA dehydrogenase deficiency (glutaric aciduria type II)]. No To Hattatsu. 2000 Mar;32(2):163-8.

23. Vergani L, Barile M, Angelini C, et al. Riboflavin therapy. Biochemical heterogeneity in two adult lipid storage myopathies. Brain. 1999 Dec;122 ( Pt 12):2401-11.

24. Mareska MC, Adams KK, Muenzer J, Frerman F, Braun TG, Howard JF, Jr. Adult-Onset Presentation of Glutaric Acidemia Type II With Myopathy. J Clin Neuromuscul Dis. 2003 Mar;4(3):124-8.

25. Uziel G, Garavaglia B, Ciceri E, Moroni I, Rimoldi M. Riboflavin-responsive glutaric aciduria type II presenting as a leukodystrophy. Pediatr Neurol. 1995 Nov;13(4):333-5.

26. Onkenhout W, Venizelos V, van der Poel PF, van den Heuvel MP, Poorthuis BJ. Identification and quantification of intermediates of unsaturated fatty acid metabolism in plasma of patients with fatty acid oxidation disorders. Clin Chem. 1995 Oct;41(10):1467-74.

27. Mooy PD, Giesberts MA, van Gelderen HH, et al. Glutaric aciduria type II: multiple defects in isolated muscle mitochondria and deficient beta-oxidation in fibroblasts. J Inherit Metab Dis. 1984;7 Suppl 2:101-2.

28. Manning NJ, Olpin SE, Pollitt RJ, Webley J. A comparison of [9,10-3H]palmitic and [9,10-3H]myristic acids for the detection of defects of fatty acid oxidation in intact cultured fibroblasts. J Inherit Metab Dis. 1990;13(1):58-68.

29. Antozzi C, Garavaglia B, Mora M, et al. Late-onset riboflavin-responsive myopathy with combined multiple acyl coenzyme A dehydrogenase and respiratory chain deficiency. Neurology. 1994 Nov;44(11):2153-8.

30. Araki E, Kobayashi T, Kohtake N, Goto I, Hashimoto T. A riboflavin-responsive lipid storage myopathy due to multiple acyl-CoA dehydrogenase deficiency: an adult case. J Neurol Sci. 1994 Nov;126(2):202-5.

31. Triggs WJ, Roe CR, Rhead WJ, Hanson SK, Lin SN, Willmore LJ. Neuropsychiatric manifestations of defect in mitochondrial beta oxidation response to riboflavin. J Neurol Neurosurg Psychiatry. 1992 Mar;55(3):209-11.

32. Mongini T, Doriguzzi C, Palmucci L, et al. Lipid storage myopathy in multiple acyl-CoA dehydrogenase deficiency: an adult case. Eur Neurol. 1992;32(3):170-6.

33. Papadimitriou A, Servidei S. Late onset lipid storage myopathy due to multiple acyl CoA dehydrogenase deficiency triggered by valproate. Neuromuscul Disord. 1991;1(4):247-52.

34. DiDonato S, Gellera C, Peluchetti D, et al. Normalization of short-chain acylcoenzyme A dehydrogenase after riboflavin treatment in a girl with multiple acylcoenzyme A dehydrogenase-deficient myopathy. Ann Neurol. 1989 May;25(5):479-84.

35. Peluchetti D, Antozzi C, Roi S, DiDonato S, Cornelio F. Riboflavin responsive multiple acyl-CoA dehydrogenase deficiency: functional evaluation of recovery after high dose vitamin supplementation. J Neurol Sci. 1991 Sep;105(1):93-8.

36. Amendt BA, Rhead WJ. The multiple acyl-coenzyme A dehydrogenation disorders, glutaric aciduria type II and ethylmalonic-adipic aciduria. Mitochondrial fatty acid oxidation, acyl-coenzyme A dehydrogenase, and electron transfer flavoprotein activities in fibroblasts. J Clin Invest. 1986 Jul;78(1):205-13.

37. Mantagos S, Genel M, Tanaka K. Ethylmalonic-adipic aciduria. In vivo and in vitro studies indicating deficiency of activities of multiple acyl-CoA dehydrogenases. J Clin Invest. 1979 Dec;64(6):1580-9.

38. Duran M, Walther FJ, Bruinvis L, Wadman SK. The urinary excretion of ethylmalonic acid: what level requires further attention? Biochem Med. 1983 Apr;29(2):171-5.

39. Rhead WJ, Wolff JA, Lipson M, et al. Clinical and biochemical variation and family studies in the multiple acyl-CoA dehydrogenation disorders. Pediatr Res. 1987 Apr;21(4):371-6.

40. Yazici N, Sarialioglu F, Alkan O, Kayaselcuk F, Erol I. Glutaric aciduria type II [corrected] and brain tumors: a case report and review of the literature. J Pediatr Hematol Oncol. 2009 Nov;31(11):865-9.

41. Law LK, Tang NL, Hui J, et al. Novel mutations in ETFDH gene in Chinese patients with riboflavin-responsive multiple acyl-CoA dehydrogenase deficiency. Clin Chim Acta. 2009 Jun 27;404(2):95-9.

42. Olsen RK, Andresen BS, Christensen E, Bross P, Skovby F, Gregersen N. Clear relationship between ETF/ETFDH genotype and phenotype in patients with multiple acyl-CoA dehydrogenation deficiency. Hum Mutat. 2003 Jul;22(1):12-23.

43. Henriques BJ, Rodrigues JV, Olsen RK, Bross P, Gomes CM. Role of flavinylation in a mild variant of multiple acyl-CoA dehydrogenation deficiency: a molecular rationale for the effects of riboflavin supplementation. J Biol Chem. 2009 Feb 13;284(7):4222-9.

44. Singla M, Guzman G, Griffin AJ, Bharati S. Cardiomyopathy in multiple Acyl-CoA dehydrogenase deficiency: a clinico-pathological correlation and review of literature. Pediatr Cardiol. 2008 Mar;29(2):446-51.

45. Lundemose JB, Kolvraa S, Gregersen N, Christensen E, Gregersen M. Fatty acid oxidation disorders as primary cause of sudden and unexpected death in infants and young children: an investigation performed on cultured fibroblasts from 79 children who died aged between 0-4 years. Mol Pathol. 1997 Aug;50(4):212-7.

46. Benigno V, Meli F, Cardella F, et al. Transient heart ischemic lesion in late onset glutaric aciduria type II (GAII). J Inherit Metab Dis. 2002;25(Suppl. 1).

47. Cotelli MS, Vielmi V, Rimoldi M, et al. Riboflavin-responsive multiple acyl-CoA dehydrogenase deficiency with unknown genetic defect. Neurol Sci. 2012 Dec;33(6):1383-7.

48. Yotsumoto Y, Hasegawa Y, Fukuda S, et al. Clinical and molecular investigations of Japanese cases of glutaric acidemia type 2. Mol Genet Metab. 2008 May;94(1):61-7.

49. Colombo I, Finocchiaro G, Garavaglia B, et al. Mutations and polymorphisms of the gene encoding the beta-subunit of the electron transfer flavoprotein in three patients with glutaric acidemia type II. Hum Mol Genet. 1994 Mar;3(3):429-35.

50. Purevjav E, Kimura M, Takusa Y, et al. Molecular study of electron transfer flavoprotein alpha-subunit deficiency in two Japanese children with different phenotypes of glutaric acidemia type II. Eur J Clin Invest. 2002 Sep;32(9):707-12.

51. Lan MY, Fu MH, Liu YF, et al. High frequency of ETFDH c.250G>A mutation in Taiwanese patients with late-onset lipid storage myopathy. Clin Genet. 2010 Dec;78(6):565-9.

52. Er TK, Liang WC, Chang JG, Jong YJ. High resolution melting analysis facilitates mutation screening of ETFDH gene: applications in riboflavin-responsive multiple acyl-CoA dehydrogenase deficiency. Clin Chim Acta. 2010 May 2;411(9-10):690-9.

53. Liang WC, Ohkuma A, Hayashi YK, et al. ETFDH mutations, CoQ10 levels, and respiratory chain activities in patients with riboflavin-responsive multiple acyl-CoA dehydrogenase deficiency. Neuromuscul Disord. 2009 Mar;19(3):212-6.

54. Liang WC, Tsai KB, Lai CL, Chen LH, Jong YJ. Riboflavin-responsive glutaric aciduria type II with recurrent pancreatitis. Pediatr Neurol. 2004 Sep;31(3):218-21.

55. Pollard LM, Williams NR, Espinoza L, et al. Diagnosis, treatment, and long-term outcomes of late-onset (type III) multiple acyl-CoA dehydrogenase deficiency. J Child Neurol. 2010 Aug;25(8):954-60.

56. Yang Y, Yao Z, Song J, et al. Outcome of organic acidurias in China. Ann Acad Med Singapore. 2008 Dec;37(12 Suppl):120-3.

57. Angle B, Burton BK. Risk of sudden death and acute life-threatening events in patients with glutaric acidemia type II. Mol Genet Metab. 2008 Jan;93(1):36-9.

58. Olsen RK, Olpin SE, Andresen BS, et al. ETFDH mutations as a major cause of riboflavin-responsive multiple acyl-CoA dehydrogenation deficiency. Brain. 2007 Aug;130(Pt 8):2045-54.

59. Gregersen N, Wintzensen H, Christensen SK, Christensen MF, Brandt NJ, Rasmussen K. C6-C10-dicarboxylic aciduria: investigations of a patient with riboflavin responsive multiple acyl-CoA dehydrogenation defects. Pediatr Res. 1982 Oct;16(10):861-8.

60. Gregersen N, Christensen MF, Christensen E, Kolvraa S. Riboflavin responsive multiple acyl-CoA dehydrogenation deficiency. Assessment of 3 years of riboflavin treatment. Acta Paediatr Scand. 1986 Jul;75(4):676-81.

61. Henderson MJ, Evans C, Patterson A, et al. Late presenting riboflavin responsive multiple acyl-CoA dehydrogenase deficiency with unusual features. J Inherit Metab Dis. 2002;25(Suppl. 1).

62. Beresford MW, Pourfarzam M, Turnbull DM, Davidson JE. So doctor, what exactly is wrong with my muscles? Glutaric aciduria type II presenting in a teenager. Neuromuscul Disord. 2006 Apr;16(4):269-73.

63. Stojanovic N, Walker V, Gatling W, Coppini DV. MADD or drunk? Adults have inborn errors too. Hosp Med. 2000 Mar;61(3):212-3.

64. Gempel K, Topaloglu H, Talim B, et al. The myopathic form of coenzyme Q10 deficiency is caused by mutations in the electron-transferring-flavoprotein dehydrogenase (ETFDH) gene. Brain. 2007 Aug;130(Pt 8):2037-44.

65. Horvath R, Schneiderat P, Schoser BG, et al. Coenzyme Q10 deficiency and isolated myopathy. Neurology. 2006 Jan 24;66(2):253-5.

66. Firat AK, Karakas HM, Yakinci C. Magnetic resonance spectroscopic characteristics of glutaric aciduria type II. Dev Med Child Neurol. 2006 Oct;48(10):847-50.

67. Schiff M, Froissart R, Olsen RK, Acquaviva C, Vianey-Saban C. Electron transfer flavoprotein deficiency: functional and molecular aspects. Mol Genet Metab. 2006 Jun;88(2):153-8.

68. Gianazza E, Vergani L, Wait R, et al. Coordinated and reversible reduction of enzymes involved in terminal oxidative metabolism in skeletal muscle mitochondria from a riboflavin-responsive, multiple acyl-CoA dehydrogenase deficiency patient. Electrophoresis. 2006 Mar;27(5-6):1182-98.

69. Olsen RK, Andresen BS, Christensen E, et al. DNA-based prenatal diagnosis for severe and variant forms of multiple acyl-CoA dehydrogenation deficiency. Prenat Diagn. 2005 Jan;25(1):60-4.

70. Russell AP, Schrauwen P, Somm E, et al. Decreased fatty acid beta-oxidation in riboflavin-responsive, multiple acylcoenzyme A dehydrogenase-deficient patients is associated with an increase in uncoupling protein-3. J Clin Endocrinol Metab. 2003 Dec;88(12):5921-6.

71. Van Hove JL, Grunewald S, Jaeken J, et al. D,L-3-hydroxybutyrate treatment of multiple acyl-CoA dehydrogenase deficiency (MADD). Lancet. 2003 Apr 26;361(9367):1433-5.

72. Loehr JP, Goodman SI, Frerman FE. Glutaric acidemia type II: heterogeneity of clinical and biochemical phenotypes. Pediatr Res. 1990 Mar;27(3):311-5.

73. Abdenur JE, Chamoles NA, Schenone AB, et al. Multiple acyl-CoA-dehydrogenase deficiency (MADD): use of acylcarnitines and fatty acids to monitor the response to dietary treatment. Pediatr Res. 2001 Jul;50(1):61-6.

74. Monici MC, Toscano A, Girlanda P, Aguennouz M, Musumeci O, Vita G. Apoptosis in metabolic myopathies. Neuroreport. 1998 Jul 13;9(10):2431-5.

75. Burns SP, Holmes HC, Chalmers RA, Johnson A, Iles RA. Proton NMR spectroscopic analysis of multiple acyl-CoA dehydrogenase deficiency--capacity of the choline oxidation pathway for methylation in vivo. Biochim Biophys Acta. 1998 Apr 28;1406(3):274-82.

76. Pang CP, Law LK, Mak YT, et al. Biochemical investigation of young hospitalized Chinese children: results over a 7-year period. Am J Med Genet. 1997 Nov 12;72(4):417-21.

77. Jakobs C, Kneer J, Martin D, et al. In vivo stable isotope studies in three patients affected with mitochondrial fatty acid oxidation disorders: limited diagnostic use of 1-13C fatty acid breath test using bolus technique. Eur J Pediatr. 1997 Aug;156 Suppl 1:S78-82.

78. Poll-The BT, Bonnefont JP, Ogier H, et al. Familial hypoketotic hypoglycaemia associated with peripheral neuropathy, pigmentary retinopathy and C6-C14 hydroxydicarboxylic aciduria. A new defect in fatty acid oxidation? J Inherit Metab Dis. 1988;11 Suppl 2:183-5.

79. Fontaine M, Briand G, Vallee L, et al. Acylcarnitine removal in a patient with acyl-CoA beta-oxidation deficiency disorder: effect of L-carnitine therapy and starvation. Clin Chim Acta. 1996 Aug 30;252(2):109-22.

80. Vallee L, Fontaine M, Nuyts JP, et al. Stroke, hemiparesis and deficient mitochondrial beta-oxidation. Eur J Pediatr. 1994 Aug;153(8):598-603.

81. Poorthuis BJ, Jille-Vlckova T, Onkenhout W. Determination of acylcarnitines in urine of patients with inborn errors of metabolism using high-performance liquid chromatography after derivatization with 4'-bromophenacylbromide. Clin Chim Acta. 1993 Jul 16;216(1-2):53-61.

82. Guffon N, Vianey-Saban C, Berthier JC, et al. [Multiple acyl-CoA dehydrogenase deficiency. Report of 2 siblings]. Pediatrie. 1993;48(5):365-71.

83. Gilkeson GS, Caldwell DS. Riboflavin-responsive multiple acyl coenzyme A dehydrogenase deficiency presenting as a proximal myopathy in a young adult. Arthritis Rheum. 1988 May;31(5):695-6.

84. al-Essa MA, Rashed MS, Bakheet SM, Patay ZJ, Ozand PT. Glutaric aciduria type II: observations in seven patients with neonatal- and late-onset disease. J Perinatol. 2000 Mar;20(2):120-8.

85. Ishii K, Komaki H, Ohkuma A, Nishino I, Nonaka I, Sasaki M. Central nervous system and muscle involvement in an adolescent patient with riboflavin-responsive multiple acyl-CoA dehydrogenase deficiency. Brain Dev. 2010 Sep;32(8):669-72.

86. Wen B, Dai T, Li W, et al. Riboflavin-responsive lipid-storage myopathy caused by ETFDH gene mutations. J Neurol Neurosurg Psychiatry. 2010 Feb;81(2):231-6.

87. Williams SF, Alvarez JR, Pedro HF, Apuzzio JJ. Glutaric aciduria type II and narcolepsy in pregnancy. Obstet Gynecol. 2008 Feb;111(2 Pt 2):522-4.

88. Dusheiko G, Kew MC, Joffe BI, Lewin JR, Mantagos S, Tanaka K. Recurrent hypoglycemia associated with glutaric aciduria type II in an adult. N Engl J Med. 1979 Dec 27;301(26):1405-9.

89. de Visser M, Scholte HR, Schutgens RB, et al. Riboflavin-responsive lipid-storage myopathy and glutaric aciduria type II of early adult onset. Neurology. 1986 Mar;36(3):367-72.

90. Tamaoki Y, Kimura M, Hasegawa Y, Iga M, Inoue M, Yamaguchi S. A survey of Japanese patients with mitochondrial fatty acid beta-oxidation and related disorders as detected from 1985 to 2000. Brain Dev. 2002 Oct;24(7):675-80.

91. Ohkuma A, Noguchi S, Sugie H, et al. Clinical and genetic analysis of lipid storage myopathies. Muscle Nerve. 2009 Mar;39(3):333-42.

92. Harpey JP, Charpentier C, Coude M, Divry P, Paturneau-Jouas M. Sudden infant death syndrome and multiple acyl-coenzyme A dehydrogenase deficiency, ethylmalonic-adipic aciduria, or systemic carnitine deficiency. J Pediatr. 1987 Jun;110(6):881-4.

93. Curcoy A, Olsen RK, Ribes A, et al. Late-onset form of beta-electron transfer flavoprotein deficiency. Mol Genet Metab. 2003 Apr;78(4):247-9.

94. Maillart E, Acquaviva-Bourdain C, Rigal O, et al. [Multiple acyl-CoA dehydrogenase deficiency (MADD): a curable cause of genetic muscular lipidosis]. Rev Neurol (Paris). 2010 Mar;166(3):289-94.

95. Green A, Marshall TG, Bennett MJ, Gray RG, Pollitt RJ. Riboflavin-responsive ethylmalonic-adipic aciduria. J Inherit Metab Dis. 1985;8(2):67-70.

96. Green A, Preece MA, de Sousa C, Pollitt RJ. Possible deleterious effect of L-carnitine supplementation in a patient with mild multiple acyl-CoA dehydrogenation deficiency (ethylmalonic-adipic aciduria). J Inherit Metab Dis. 1991;14(5):691-7.

97. Xi J, Wen B, Lin J, et al. Clinical features and ETFDH mutation spectrum in a cohort of 90 Chinese patients with late-onset multiple acyl-CoA dehydrogenase deficiency. J Inherit Metab Dis. 2013 Dec 20.

98. Scheicht D, Werthmann ML, Zeglam S, Holtmeier J, Holtmeier W, Strunk J. [Muscle weakness and early stages of liver failure in a 22-year-old man]. Internist (Berl). 2013 Aug;54(8):1016-22.

99. Chien YH, Lee NC, Chao MC, et al. Fatty Acid Oxidation Disorders in a Chinese Population in Taiwan. JIMD Rep. 2013 May 23.

100. Rosenbohm A, Sussmuth SD, Kassubek J, et al. Novel ETFDH mutation and imaging findings in an adult with glutaric aciduria type II. Muscle Nerve. 2013 Jul 28.

101. Wen B, Li D, Shan J, et al. Increased muscle coenzyme Q10 in riboflavin responsive MADD with ETFDH gene mutations due to secondary mitochondrial proliferation. Mol Genet Metab. 2013 Jun;109(2):154-60.
